# Identifying gaps for research prioritisation: Global burden of external causes of injury as reflected in the *Cochrane Database of Systematic Reviews*^☆^

**DOI:** 10.1016/j.injury.2015.12.019

**Published:** 2016-05

**Authors:** Chante Karimkhani, Ritika Trikha, Baran Aksut, Trevor Jones, Lindsay N. Boyers, Megan Schlichte, Hannah Pederson, Tyler Okland, Carolyn DiGuiseppi, Mona Nasser, Mohsen Naghavi, Theo Vos, Sze Lin Yoong, Luke Wolfenden, Christopher J.L. Murray, Robert P. Dellavalle

**Affiliations:** a Columbia University College of Physicians and Surgeons, New York, NY, USA; b Rosalind Franklin University of Medicine and Science, North Chicago, IL, USA; c Columbia University Medical Center, New York, NY, USA; d University of Arizona College of Medicine, Tucson, AZ, USA; e Georgetown University School of Medicine, Washington DC, USA; f Baylor College of Medicine, Houston, TX, USA; g University of Colorado School of Medicine, Aurora, CO, USA; h Department of Epidemiology, Colorado School of Public Health, University of Colorado Anschutz Medical Campus, CO, USA; i Peninsula Dental School, Plymouth University, Plymouth, UK; j Institute for Health Metrics and Evaluation, University of Washington, Seattle, WA, USA; k Hunter New England Population Health, NSW, Australia; l The University of Newcastle, NSW, Australia; m Department of Dermatology, University of Colorado Anschutz Medical Campus, CO, USA; n Department of Dermatology, Denver Veterans Administration Hospital, CO, USA

**Keywords:** Burden of disease, DALY, Epidemiology, Injury, Trauma, Disability, Systematic review, Cochrane

## Abstract

**Importance:**

Burden of disease should impact research prioritisation.

**Objective:**

To analyse the *Cochrane Database of Systematic Reviews* (CDSR) and determine whether systematic reviews and protocols accurately represent disease burden, as measured by disability-adjusted life years (DALYs) from the Global Burden of Disease (GBD) 2010 Study.

**Methods:**

Two investigators collected GBD disability metrics for 12 external causes of injury in the GBD 2010 Study. These external causes were then assessed for systematic review and protocol representation in CDSR. Data was collected during the month of April 2015. There were no study participants aside from the researchers. Percentage of total 2010 DALYs, 2010 DALY rank, and median DALY percent change from 1990 to 2010 of the 12 external causes of injury were compared with CDSR representation of systematic reviews and protocols. Data were analysed for correlation using Spearman rank correlation.

**Results:**

Eleven of the 12 causes were represented by at least one systematic review or protocol in CDSR; the category *collective violence and legal intervention* had no representation in CDSR. Correlation testing revealed a strong positive correlation that was statistically significant. Representation of *road injury*; *interpersonal violence*; *fire, heat, and hot substances*; *mechanical forces*; *poisonings*, *adverse effect of medical treatment*, and *animal contact* was well aligned with respect to DALY. Representation of *falls* was greater compared to DALY, while *self-harm*, *exposure to forces of nature*, and *other transport injury* representation was lower compared to DALY.

**Conclusions and relevance:**

CDSR representation of external causes of injury strongly correlates with disease burden. The number of systematic reviews and protocols was well aligned for seven out of 12 causes of injury. These results provide high-quality and transparent data that may guide future prioritisation decisions.

## Introduction

Modern research is driven by many different incentives and motivations, making the allocation of available funding quite limited [1]. It is the duty of current health practitioners to pursue research endeavours based on sound data that reflects the need for such research [2]. Priorities must be set in place to guide practitioners towards conducting meaningful research that will benefit society. Cost is one of the major driving forces behind research, while other prioritisation criteria include lack of economical interventions, opportunity for pioneering therapies, prevention of highly transmissible diseases, public interest, and burden of disease [1], [2], [3].

Few efforts take place to drive agendas and prioritisation of research specifically within the field of injuries and trauma. For example, a research agenda for geriatric emergency medicine was proposed by the Academy of Emergency Medicine [4]. The agenda authors sought to gather data on articles pertaining to trauma, using search terms of *penetrating wounds* and *non-penetrating wounds*. The goal was to provide a synthesis of current literature and suggest areas for further research. Furthermore, the Fogarty International Center of the National Institutes of Health assembled a global panel of trauma and injury experts to identify research and technological advancement needs to reduce the burden of trauma and injuries in low- and middle-income countries [5]. While both of the described efforts strove to develop research agendas and prioritisation, neither takes into account the impact of injuries and trauma on particular populations. We hope to expand upon these efforts by providing transparent data on the global burden of injuries and trauma with corresponding systematic review representation.

The Global Burden of Disease (GBD) 2010 Study is a systematic assessment of the disability and mortality of major diseases and risk factors worldwide [6]. It is a collaborative effort of scientists and researchers from the World Health Organization (WHO), World Bank, Institute for Health Metrics and Evaluation (IHME), Harvard School of Public Health, and University of Auckland School of Population Health. GBD 2010 estimates the burden of 291 diseases and injuries in 187 countries from 1990 to 2010 [7]. The disability-adjusted life year (DALY) was developed as a standardised metric to compare burden across various disease states. The DALY metric is composed of years of life lost (YLL) due to disease-causing mortality and years lived with disability (YLD) [8]. This provides high-quality epidemiological data on health status that is independent of interest groups. GBD 2010 serves as a universal measurement to inform research output and determine efficacious and cost-effective interventions. DALY metrics are estimated for 12 external causes of injury based on prevalence and data availability.

Systematic reviews are crucial to evidence-based medicine and the reduction of disease burden in the community. By providing a summary of the highest-quality, current literature relevant to a research topic, systematic reviews are increasingly recognised as the pillar of research translation [9]. Topic coverage in high-quality systematic reviews may serve as a proxy of research prioritisation. The Cochrane Collaboration is a group of 31,000 healthcare specialists whose purpose is to establish interventions and protocols of evidence-based medicine to assist healthcare professionals in clinical decision-making [10], [11]. The *Cochrane Database of Systematic Reviews* (CDSR) consists of systematic reviews and protocols (proposals for future systematic reviews) covering a diversity of diagnostic, therapeutic, and epidemiological topics. As the gold standard of systematic reviews, Cochrane reviews represent the most current evidence to ensure applicability to health-care practitioners and organisations around the world [10], [11]. However, information regarding the impact of disease burden on priority-setting of CDSR is lacking. This study will examine whether CDSR representation of 12 external causes of injury correlates with their respective disease burden as measured by GBD 2010.

## Methods

The following 12 external causes of injury were analysed in GBD 2010: road injury; other transport injuries; falls; fire, heat, and hot substances; poisonings; mechanical forces; adverse effects of medical treatment; animal contact; self-harm; interpersonal violence; exposure to forces of nature; collective violence and legal intervention. The category of other transport injuries includes injuries caused by watercrafts, airways, ships, airplanes, and railways. Keywords and synonyms from ICD-10 code definitions, as defined by GBD 2010, were used to generate search terms for each of the 12 causes. Search terms were entered into the CDSR “title, abstract, keywords” search function [12]. Upon reviewing search results within CDSR, systematic reviews and protocols were matched to a corresponding injury or trauma based on subject content and study objectives. In order to be included, a review or protocol must have included the particular external cause of injury as a primary outcome and predominant focus of the subject content, study objectives, and results. Data was also collected on type of publication (systematic review or protocol), Cochrane review group responsible for publication, date of online publication, and the number of trials included in each systematic review. Two authors (CK and BA) collected data independently in February 2015 with final consensus during April 2015. Institutional board review approval was not required since the study solely used data in the public domain and no living subjects.

Methods used by GBD 2010 to measure DALY metrics have been previously described and are available for public access [6], [13], [14]. The following two DALY metrics were collected for each of the 12 external causes of injury: percent of total 2010 DALYs (of all 291 diseases studied by GBD) and 2010 DALY rate per 100,000 persons (see Table 1). CDSR representation and DALY metrics were compared using a Spearman rank correlation. Rho, a measure of correlation was determined, along with a two-tailed *p*-value, which tests the null hypothesis of no correlation. In addition, a data plot was created with the number of review and protocol titles and percent of total 2010 DALYs for each of the 12 external causes of injury to generate a line of best fit with a coefficient of determination (*R*^2^). The trend line allowed for spatial demonstration of over- or under-representation of injuries and trauma in CDSR in relation to disease burden.

## Results

Search terms for the 12 external causes of injury yielded a total of 646 review and protocol titles, of which 459 were ultimately excluded due to lack of abstract content, objectives, or results focus on the particular cause of injury (see Table 2). A compiled list of all included and excluded reviews and protocols for the 12 external causes of injury can be found in eTables 1 and 2. It should be noted that the Cochrane Library search function provides a list of all review and protocol titles that include a particular search term at least once in the abstract. For this reason, the use of general search terms such as, ‘fall’ and ‘car’ yielded extraneous titles that were irrelevant and excluded. The overall proportion of retained initial ‘hits’ was 29% and ranged from 0% for *collective violence and legal intervention* to 78% for *interpersonal violence*.

Supplementary material related to this article can be found, in the online version, at http://dx.doi.org/10.1016/j.injury.2015.12.019.



A total of 187 reviews and protocols were included to represent the 12 external causes of injury. The Cochrane Injuries Group published the majority of the titles (*n* = 115). Other Cochrane Groups that contributed to CDSR representation of the 12 causes are as follows: Bone, Joint and Muscle Trauma Group (30); Developmental, Psychosocial and Learning Problems Group (15); Wounds Group (9); Musculoskeletal Group (7); Depression, Anxiety, and Neurosis Group (6); Oral Health Group (5); Eyes and Vision Group (4); Stroke Group (3); Anaesthesia Group (3); HIV/AIDS Group (3); Neonatal Group (2); Dementia and Cognitive Improvement Group (2); Back Group (2); Pain, Palliative, and Supportive Group (2); Drugs and Alcohol Group (2); Public Health Group (1); Drugs and Alcohol Group (1); Peripheral Vascular Diseases Group (1); Hypertension Group (1); Hepato-Biliary Group (1); Effective Practice and Organisation of Care Group (1); Renal Group (1); Occupational Safety and Health Group (1); Childhood Cancer Group (1); Pregnancy and Childbirth Group (1); Incontinence Group (1); Infectious Diseases Group (1); Skin Group (1); Gynaecological Group (1). The majority of reviews and protocols covering the 12 causes of injury were published in 2000–2010 (*n* = 133), followed by 2011 to 2015 (94). Only 4 reviews and protocols were published prior to 2000.

**Fig. 1:**
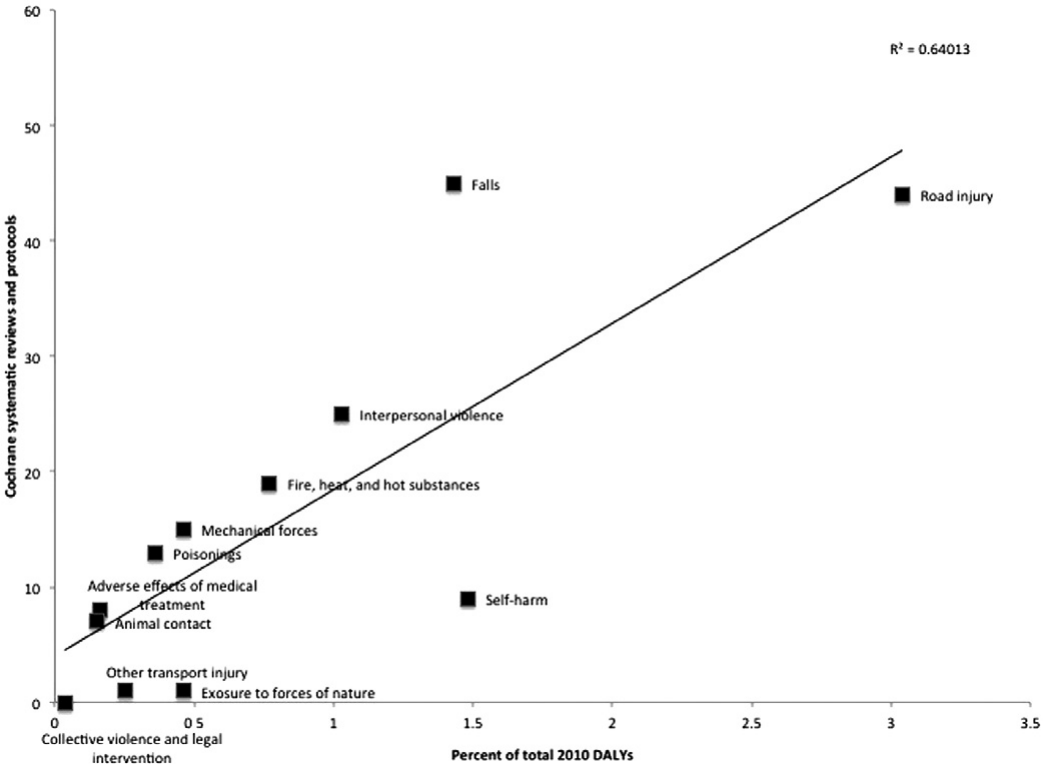
Relationship between number of systematic reviews and protocols in The Cochrane Database of Systematic Reviews and percent of total 2010 DALYs for 12 injury and trauma conditions.

*Falls* and *road injury* had the greatest representation in CDSR with 45 and 44 titles, respectively (see Table 2 and Fig. 1). These two causes also had the greatest cumulative number of studies informing the systematic review evidence-base (911 and 515, respectively). *Falls* had disproportionately greater CDSR representation compared to its disease burden.

Correlation testing between DALY and CDSR title representation demonstrated rho = 0.77, two-tailed *p* = 0.003, indicating a strong positive correlation that was statistically significant. CDSR representation of *road injury*; *interpersonal violence*; *fire, heat, and hot substances*; *mechanical forces*; *poisonings*, *adverse effect of medical treatment*, and *animal contact* was well aligned with DALY metrics. Representation of *falls* was greater than the corresponding DALY metric, while *self-harm*, *exposure to forces of nature*, and *other transport injury* representation fell below the corresponding DALY metrics (see Fig. 1).

## Discussion

Eleven of the 12 external causes of injury analysed in GBD 2010 were represented by at least one review or protocol in CDSR. *Collective violence and legal intervention*, which includes violence between nations, states, terror groups, or gangs such as war, was not matched with a review or protocol in CDSR. The results of our statistical analysis demonstrate a strong positive correlation between representation of causes of injury in CDSR and respective disease burden. This may indicate that prioritisation efforts by Cochrane review groups, particularly the Injuries Group responsible for the majority of publications, strongly considers disease burden when setting research priorities. The GBD database is becoming the pre-eminent global epidemiological data source [15]. Findings reported here are consistent with reports of weak association between global burden of disease and the number of published randomised trials [16] and moderate correlation between systematic reviews and DALYS across the entire Cochrane Database of Systematic Reviews [17]. In addition, most reviews and protocols from our analysis were up-to-date with all but four published after 2000 and 50% in the last five years. The availability of systematic reviews that summarise the most current literature is paramount for clinical decision-making.

### Conditions for which CDSR representation was greater than disease burden

*Falls* was the only cause of injury with CDSR representation that exceeded its disease burden. Out of all 291 conditions studied by GBD 2010, falls are responsible for 1.43% of disease burden. Falls are an important clinical presentation with an array of aetiologies and consequences involving multiple medical specialties such as internal medicine, geriatrics, orthopaedic surgery, neurology, and stroke services. This diversity was reflected in the Cochrane review groups responsible for titles covering *falls*: Bone, Joint and Muscle Trauma Group (*n* = 24); Musculoskeletal Group (7); Injuries Group: 6; Stroke Group (3); Eyes and Vision Group (2); Oral Health Group (1); Hypertension Group (1); and Back Group (1). This array of specialties yielded reviews covering diverse aspects of falls including prevention, surgical intervention, special considerations in the elderly population, and adverse events such as traumatic brain injury, rehabilitation, and osteoporosis.

### Conditions for which CDSR representation was lower than disease burden

*Self-harm* had the second greatest disease burden of the 12 causes of injury (1.48%), but the fourth lowest number of systematic reviews and protocols (5). *Exposure to forces of nature* (such as heat wave, earthquake, blizzard, and tornado) and *other transport injuries* (injuries due to watercrafts, aircrafts, and railways) each had only one review in CDSR. The one review on *other transport injuries*, which examined DVT prophylaxis in airline passengers, was based on 10 studies, while the review on *exposure to forces of nature*, which attempted to examine the impact of electric fans during heatwaves, found no published or un-published studies in the literature on this topic. Clearly, there is a paucity of high-quality data from the medical research community on these potentially catastrophic conditions.

### Limitations and future directions

There is some degree of subjectivity in assigning each systematic review or protocol to a particular cause or condition, which we attempted to address with data collection by two independent authors. CDSR provides systematic reviews and protocols based on published and unpublished literature meeting pre-specified eligibility criteria for a particular topic, making meta-analyses impossible for certain topics which do not contain research-based evidence in the literature [18]. Furthermore, a systematic review may synthesise many interventions (“lumping”) or be divided into several different reviews of individual interventions (“splitting”). This was especially applicable for several reviews published by the Cochrane Injuries Group, such as “Home safety education and provision of safety equipment for injury prevention” which covered *fire, heat and hot substances*; *poisonings*; *mechanical forces*; and *interpersonal violence* and was assigned to all four causes of injury.

This study is part of a larger series intended to map all 291 diseases studied by GBD to representation in major research databases [19], [20], [21], [22]. It would also be valuable to compare topic prioritisation in other databases to CDSR, such as the Database of Abstracts and Reviews of Effects (DARE), Medline, Health Systems Evidence, and Web of Science, to provide further insight into whether research priorities are appropriately matched to corresponding disease burden.

## Conclusions

Clinicians and researchers rely on databases such as the Cochrane Library, to inform the public for the prevention and treatment of injury. Thus, prioritisation methodology of major research databases is directly relevant to clinicians and researchers alike.

Disease burden should be considered in research prioritisation. Established criteria have been identified by the Cochrane Collaboration to guide prioritisation efforts regarding decisions to register a new title or update an existing review [23]. Many variables play a part in research prioritisation including cost, disease transmissibility, vulnerability of populations, and public interest. There is a lack of transparency in many organisations regarding criteria used to drive prioritisation decisions. This study sought to provide transparent data evaluating priority setting of Cochrane systematic reviews. Clinicians see the impact of injury at the individual patient level on a daily basis. The GBD Study provides both detailed view of the burden of injury and a novel tool for injury research prioritisation.

## Funding

This study was supported in part by: This project received direct funding from the Bill and Melinda Gates Foundation (PI: Christopher J.L. Murray). Lindsay Boyers and Robert Dellavalle are employees of the U.S. Department of Veterans Affairs. The U.S. Department of Veterans Affairs had no role in the design and execution of the study. Any opinions expressed herein do not necessarily reflect the opinions of the U.S. Department of Veterans Affairs.

## Conflict of interest

None declared.

## Figures and Tables

**Table 1:** Twelve external causes of injury studied by GBD 2010 with percent of total DALYs and 2010 DALY rate per 100,000 persons (arranged in order of decreasing % of total DALY).

Condition	% Total 2010 DALYs (out of 291 conditions)	2010 DALY rate per 100,000 persons
Road injury	3.04	1096
Other transport injury	0.25	88
Falls	1.43	514
Fire, heat, and hot substances	0.77	276
Mechanical forces	0.46	165
Poisonings	0.36	130
Adverse effects of medical treatment	0.16	59
Animal contact	0.15	53
Self-harm	1.48	532
Interpersonal violence	1.03	371
Exposure to forces of nature	0.46	194
Collective violence and legal intervention	0.035	14

**Table 2:** Twelve external causes of injury studied by GBD 2010 with corresponding ICD-10 codes, search terms, number of systematic reviews (R) and protocols (P) in CDSR, and number of studies in Cochrane review.

Condition	ICD-10 code	Search terms	Number OF Cochrane Reviews (R) and protocols (P)	Number of studies in Cochrane Review
Road injury	V01-V04, V06, V09, V10-V19, V20-V29, V30-V79, V87.2-V87.3, V80, V82, V05, V81, V83-V86, V88.2, V88.3, V91, V93-V98,	“pedal cycle”	40 (R) 4 (P)	515
“bicycle”
“bus”
“car”
“truck”
“van”
“vehicle”
“roller-skates”
“pedestrian collision”
“pedestrian injury”
“skateboard”
“transport accident”
“animal drawn vehicle”
“streetcar”
“pedal cycle”
“bicyclist”
“motorcycle”
“moped”
“sidecar”
“motorised bicycle”
“motor scooter”
“three-wheeled”
“car occupant”
“pick-up truck”
“van”
“heavy transport vehicle”
“bus”
“minibus”
“motorised tricycle”
“traffic”
“animal rider”
“horse rider”
“railway”
“streetcar”
“animal-drawn”

Other transport injury	V05, V81, V83-V86, V88.2, V88.3, V91, V93-V98	“pedestrian railway”	1 (R)	10
“person car pick-up truck”
“person car van”
“person car bus”
“watercraft”
“aircraft”
“vehicle”
“non-traffic collision”
“transport accident”
“ship”
“waterskiing”
“helicopter”
“ultralight”
“microlight”
“powered glider”
“spacecraft”
“balloon accident”
“hang-glider”
“air transport”
“cable car”
“yacht”
“ski-lift”

Falls	W00-W19	“fall”	44 (R) 1 (P)	911

Fire, heat, and hot substances	X00-X19	“fire”	19 (R)	246
“ignition”
“flame”
“steam”
“burning building”
“gasoline”
“burn”

Mechanical forces	W32-W34, W24-W31, W45-W46, W21, W39, W44, W49-W52, W75-W99, X50-X58	“handgun”	15 (R)	314
“rifle”
“shotgun”
“firearm”
“lifting devices”
“sharp glass”
“knife”
“hand tool”
“lawnmower”
“machinery”
“foreign body”
“nail skin”
“contact needle”
“stab”
“sports equipment”
“firework”
“gravitational forces”
“hit”
“strike”
“kick”
“bite”
“scratch”
“stampede”
“suffocation”
“aspiration of gastric contents”
“accidental asphyxia”
“choke”
“electric line”
“electric current”
“electric shock”
“electrocution”
“mountain sickness”
“insufficient nourishment”
“starvation”
“destitution”
“drowning”

Poisonings	X40, X43-X44 X46-X48	“poisoning”	11 (R) 2 (P)	194

Adverse effects of medical treatment	Y40-Y84, Y88	“unintentional cut”	7 (R) 1 (P)	45
“unintentional puncture”
“unintentional haemorrhage”
“sterile precautions”
“overmedication”
“undermedication”
“contaminated medical”
“contaminated biological”
“blood mismatch”
“nonadministration”
“malfunction”
“medical devices”
“adverse medical”

Animal contact	X20-X29, W53-W64	“snake”	6 (R) 1 (P)	26
“lizard”
“fer de lance”
“gila monster”
“krait”
“spider”
“scorpion”
“hornet”
“wasp”
“bee”
“yellow jacket”
“centipede”
“millipede”
“ant”
“caterpillar”
“arthropod”
“venom”
“rodent”
“sting”
“squirrel”
“dog”
“marine animal”
“alligator”
“crocodile”
“reptile”
“bird”
“amphibian”
“lizard”
“thorn”

Self-harm	X60-X83	“self-harm”	6 (R) 3 (P)	35
“self-poisoning”
“suicide”

Interpersonal violence	X93-X95, X99, X85-X92, X96-X98, Y00-Y08	“assault”	22 (R) 3 (P)	338
“homicidal poisoning”
“drowning”
“explosive”
“rape”
“sexual assault”
“sodomy”
“maltreatment”
“mistreatment”
“mental cruelty”
“physical abuse”


Exposure to forces of nature	X30-X39	“heat stroke”	1 (R)	0
“heatwave”
“earthquake”
“volcano”
“tsunami”
“avalanche”
“landslide”
“mudslide”
“cataclysmic storm”
“blizzard”
“cloudburst”
“cyclone”
“hurricane”
“tidal wave”
“tornado”
“torrential rain”
“flood”

Collective violence and legal intervention	Y35-36, Y89.0, Y89.1	“collective violence”	0	0
“war”
“legal intervention”
“legal execution”
