# Supplementary material for: Identifying gaps for research prioritisation: Global burden of external causes of injury as reflected in the Cochrane Database of Systematic Reviews
Source: Injury. 2016 May;47(5):1151–7. doi: 10.1016/j.injury.2015.12.019 (PMC4862959; doi:10.1016/j.injury.2015.12.019)
Supplement: Supplementary file 1 [file mmc1.docx]

**eTable 1: Titles for all reviews and protocols included for injuries and trauma studied by GBD 2010 and associated Cochrane Group responsible for publication**

| **Injury or Trauma** | **Review (R) or Protocol (P) Title** | **Cochrane Group** | **Number of Studies** | **Year of Online Publication** |
| --- | --- | --- | --- | --- |
| Road Injury | [**Bicycle** helmet legislation for the uptake of helmet use and prevention of head injuries](http://onlinelibrary.wiley.com/doi/10.1002/14651858.CD005401.pub3/abstract) (R) | Cochrane Injuries Group | 6 | 2008 |
|  | [Pre-discharge "**car** seat challenge" for preventing morbidity and mortality in preterm infants](http://onlinelibrary.wiley.com/doi/10.1002/14651858.CD005386.pub2/abstract) (R) | Cochrane Neonatal Group | 0 | 2006 |
|  | [Helmets for preventing head and facial injuries in bicyclists](http://onlinelibrary.wiley.com/doi/10.1002/14651858.CD001855/abstract) (R) | Cochrane Injuries Group | 5 | 1999 |
|  | [Organisational travel plans for improving health](http://onlinelibrary.wiley.com/doi/10.1002/14651858.CD005575.pub3/abstract) (R) | Cochrane Injuries Group | 17 | 2010 |
|  | [Interventions for the management of mandibular fractures](http://onlinelibrary.wiley.com/doi/10.1002/14651858.CD006087.pub3/abstract) (R) | Cochrane Oral Health Group | 12 | 2013 |
|  | [Interventions for increasing pedestrian and cyclist visibility for the prevention of death and injuries](http://onlinelibrary.wiley.com/doi/10.1002/14651858.CD003438.pub2/abstract) (R) | Cochrane Injuries Group | 42 | 2006 |
|  | [Non-legislative interventions for the promotion of cycle helmet wearing by children](http://onlinelibrary.wiley.com/doi/10.1002/14651858.CD003985.pub3/abstract) (R) | Cochrane Injuries Group | 29 | 2011 |
|  | [Alcohol and drug screening of occupational drivers for preventing injury](http://onlinelibrary.wiley.com/doi/10.1002/14651858.CD006566.pub2/abstract) (R) | Cochrane Injuries Group | 2 | 2009 |
|  | [Driving assessment for maintaining mobility and safety in drivers with dementia](http://onlinelibrary.wiley.com/doi/10.1002/14651858.CD006222.pub4/abstract) (R) | Cochrane Dementia and Cognitive Improvement Group | 0 | 2013 |
|  | [Alcohol ignition interlock programmes for reducing drink driving recidivism](http://onlinelibrary.wiley.com/doi/10.1002/14651858.CD004168.pub2/abstract) (R) | Cochrane Injuries Group | 11 | 2004 |
|  | [The 'WHO Safe Communities' model for the prevention of injury in whole populations](http://onlinelibrary.wiley.com/doi/10.1002/14651858.CD004445.pub3/abstract) (R) | Cochrane Injuries Group | 21 | 2009 |
|  | [Non-operative versus operative treatment for blunt pancreatic trauma in children](http://onlinelibrary.wiley.com/doi/10.1002/14651858.CD009746.pub2/abstract) (R) | Cochrane Injuries Group | 0 | 2002 |
|  | [Safety education of pedestrians for injury prevention](http://onlinelibrary.wiley.com/doi/10.1002/14651858.CD001531/abstract) (R) | Cochrane Injuries Group | 15 | 2002 |
|  | [Interventions for promoting booster seat use in four to eight year olds travelling in motor **vehicles**](http://onlinelibrary.wiley.com/doi/10.1002/14651858.CD004334.pub2/abstract) (R) | Cochrane Injuries Group | 5 | 2006 |
|  | [Graduated driver licensing for reducing motor **vehicle** crashes among young drivers](http://onlinelibrary.wiley.com/doi/10.1002/14651858.CD003300.pub3/abstract) (R) | Cochrane Injuries Group | 34 | 2011 |
|  | [Speed cameras for the prevention of road traffic injuries and deaths](http://onlinelibrary.wiley.com/doi/10.1002/14651858.CD004607.pub4/abstract) (R) | Cochrane Injuries Group | 35 | 2010 |
|  | [Driving assessment for maintaining mobility and safety in drivers with dementia](http://onlinelibrary.wiley.com/doi/10.1002/14651858.CD006222.pub4/abstract) (R) | Cochrane Dementia and Cognitive Improvement Group | 0 | 2013 |
|  | [Interventions for preventing injuries in problem drinkers](http://onlinelibrary.wiley.com/doi/10.1002/14651858.CD001857.pub2/abstract) (R) | Cochrane Injuries Group | 17 | 2004 |
|  | [Vision screening of older drivers for preventing road traffic injuries and fatalities](http://onlinelibrary.wiley.com/doi/10.1002/14651858.CD006252.pub4/abstract) (R) | Cochrane Injuries Group | 0 | 2014 |
|  | [Area-wide traffic calming for preventing traffic related injuries](http://onlinelibrary.wiley.com/doi/10.1002/14651858.CD003110/abstract) (R) | Cochrane Injuries Group | 22 | 2003 |
|  | [Motorcycle rider training for the prevention of road traffic crashes](http://onlinelibrary.wiley.com/doi/10.1002/14651858.CD005240.pub2/abstract) (R) | Cochrane Injuries Group | 23 | 2010 |
|  | `[Increased police patrols for preventing alcohol-impaired driving](http://onlinelibrary.wiley.com/doi/10.1002/14651858.CD005242.pub2/abstract) (R) | Cochrane Injuries Group | 32 | 2008 |
|  | [Alcohol and drug screening of occupational drivers for preventing work-related injury](http://onlinelibrary.wiley.com/doi/10.1002/14651858.CD010043/abstract) (P) | Cochrane Injuries Group | N/A | 2012 |
|  | [Conservative treatments for whiplash](http://onlinelibrary.wiley.com/doi/10.1002/14651858.CD003338.pub3/abstract) (R) | Cochrane Back Group | 23 | 2007 |
|  | [Surgical versus non-surgical treatment for thoracolumbar burst fractures without neurological deficit](http://onlinelibrary.wiley.com/doi/10.1002/14651858.CD005079.pub3/abstract) (R) | Cochrane Bone, Joint, and Muscle Trauma Group | 2 | 2013 |
|  | [Mobility management for prevented, reduced, or delayed driving in teenagers](http://onlinelibrary.wiley.com/doi/10.1002/14651858.CD009438/abstract) (P) | Cochrane Injuries Group | N/A | 2011 |
|  | [Helmets for preventing injury in **motorcycle** riders](http://onlinelibrary.wiley.com/doi/10.1002/14651858.CD004333.pub3/abstract) (R) | Cochrane Injuries Group | 61 | 2008 |
|  | [Increasing **motorcycle** and rider conspicuity for preventing death and injury in motorcyclists](http://onlinelibrary.wiley.com/doi/10.1002/14651858.CD004608/abstract) (P) | Cochrane Injuries Group | N/A | 2003 |
|  | [**Motorcycle** helmet legislation for preventing injuries in motorcyclists](http://onlinelibrary.wiley.com/doi/10.1002/14651858.CD004891/abstract) (P) | Cochrane Injuries Group | N/A | 2004 |
|  | [Post-licence driver education for the prevention of road **traffic** crashes](http://onlinelibrary.wiley.com/doi/10.1002/14651858.CD003734/abstract) (R) | Cochrane Injuries Group | 24 | 2003 |
|  | [Street lighting for preventing road **traffic** injuries](http://onlinelibrary.wiley.com/doi/10.1002/14651858.CD004728.pub2/abstract) (R) | Cochrane Injuries Group | 17 | 2009 |
|  | [School-based driver education for the prevention of **traffic** crashes](http://onlinelibrary.wiley.com/doi/10.1002/14651858.CD003201/abstract) (R) | Cochrane Injuries Group | 3 | 2001 |
|  | [Red-light cameras for the prevention of road **traffic** crashes](http://onlinelibrary.wiley.com/doi/10.1002/14651858.CD003862.pub2/abstract) (R) | Cochrane Injuries Group | 10 | 2005 |
|  | [Regulatory and road engineering interventions for preventing road **traffic** injuries and fatalities among vulnerable (non-motorised and motorised two-wheel) road users in low- and middle-income countries](http://onlinelibrary.wiley.com/doi/10.1002/14651858.CD011495/abstract) (P) | Cochrane Public Health Group | N/A | 2015 |
|  | [Caffeine for the prevention of injuries and errors in shift workers](http://onlinelibrary.wiley.com/doi/10.1002/14651858.CD008508/abstract) (R) | Cochrane Injuries Group | 13 | 2010 |
|  | [Psychosocial interventions for the prevention of disability following traumatic physical injury](http://onlinelibrary.wiley.com/doi/10.1002/14651858.CD006422.pub3/abstract) (R) | Cochrane Injuries Group | 5 | 2009 |
|  | [Magnesium for acute traumatic brain injury](http://onlinelibrary.wiley.com/doi/10.1002/14651858.CD005400.pub3/abstract) (R) | Cochrane Injuries Group | 3 | 2008 |
|  | [Hyperbaric oxygen therapy for the adjunctive treatment of traumatic brain injury](http://onlinelibrary.wiley.com/doi/10.1002/14651858.CD004609.pub3/abstract) (R) | Cochrane Injuries Group | 7 | 2012 |
|  | [Modest cooling therapies (35ºC to 37.5ºC) for traumatic brain injury](http://onlinelibrary.wiley.com/doi/10.1002/14651858.CD006811.pub3/abstract) (R) | Cochrane Injuries Group | 0 | 2014 |
|  | [Restricting or banning alcohol advertising to reduce alcohol consumption in adults and adolescents](http://onlinelibrary.wiley.com/doi/10.1002/14651858.CD010704.pub2/abstract) (R) | Cochrane Drugs and Alcohol Group | 4 | 2014 |
|  | [Bone grafts and bone substitutes for treating distal radial fractures in adults](http://onlinelibrary.wiley.com/doi/10.1002/14651858.CD006836.pub2/abstract) (R) | Cochrane Bone, Joint, and Muscle Trauma Group | 10 | 2008 |
|  | [Surgical approaches for cervical spine facet dislocations in adults](http://onlinelibrary.wiley.com/doi/10.1002/14651858.CD008129.pub2/abstract) (R) | Cochrane Bone, Joint, and Muscle Trauma Group | 2 | 2014 |
|  | [Gangliosides for acute spinal cord injury](http://onlinelibrary.wiley.com/doi/10.1002/14651858.CD004444.pub2/abstract) (R) | Cochrane Injuries Group | 2 | 2005 |
|  | [Surgical versus non-surgical management of abdominal injury](http://onlinelibrary.wiley.com/doi/10.1002/14651858.CD007383.pub2/abstract) (R) | Cochrane Injuries Group | 1 | 2012 |
| Other Transport Injury | Compression stockings for preventing deep vein thrombosis in airline passengers (R) | Cochrane Peripheral Vascular Diseases Group | 10 | 2006 |
| Falls | [Interventions for preventing **falls** in older people living in the community](http://onlinelibrary.wiley.com/doi/10.1002/14651858.CD007146.pub3/abstract) (R) | Cochrane Bone, Joint, and Muscle Trauma Group | 159 | 2012 |
|  | [Exercise for reducing fear of **falling** in older people living in the community](http://onlinelibrary.wiley.com/doi/10.1002/14651858.CD009848.pub2/abstract) (R) | Cochrane Bone, Joint, and Muscle Trauma Group | 30 | 2014 |
|  | [Interventions for preventing **falls** in older people in care facilities and hospitals](http://onlinelibrary.wiley.com/doi/10.1002/14651858.CD005465.pub3/abstract) (R) | Cochrane Bone, Joint, and Muscle Trauma Group | 60 | 2012 |
|  | [Interventions for preventing **falls** in people after stroke](http://onlinelibrary.wiley.com/doi/10.1002/14651858.CD008728.pub2/abstract) (R) | Cochrane Stroke Group | 10 | 2013 |
|  | [Population-based interventions for the prevention of **fall** -related injuries in older people](http://onlinelibrary.wiley.com/doi/10.1002/14651858.CD004441.pub2/abstract) (R) | Cochrane Injuries Group | 6 | 2005 |
|  | [Interventions designed to prevent healthcare bed-related injuries in patients](http://onlinelibrary.wiley.com/doi/10.1002/14651858.CD008931.pub3/abstract) (R) | Cochrane Injuries Group | 2 | 2012 |
|  | [Environmental and behavioural interventions for reducing physical activity limitation in community-dwelling visually impaired older people](http://onlinelibrary.wiley.com/doi/10.1002/14651858.CD009233.pub2/abstract) (R) | Cochrane Eyes and Vision Group | 0 | 2013 |
|  | [Interventions for improving sit-to-stand ability following stroke](http://onlinelibrary.wiley.com/doi/10.1002/14651858.CD007232.pub4/abstract) (R) | Cochrane Stroke Group | 13 | 2014 |
|  | [Hip protectors for preventing hip fractures in older people](http://onlinelibrary.wiley.com/doi/10.1002/14651858.CD001255.pub5/abstract) (R) | Cochrane Bone, Joint, and Muscle Trauma Group | 19 | 2014 |
|  | [Modification of the home environment for the reduction of injuries](http://onlinelibrary.wiley.com/doi/10.1002/14651858.CD003600.pub3/abstract) (R) | Cochrane Injuries Group | 28 | 2011 |
|  | [Interventions for preventing injuries in problem drinkers](http://onlinelibrary.wiley.com/doi/10.1002/14651858.CD001857.pub2/abstract) (R) | Cochrane Injuries Group | 17 | 2004 |
|  | [Impact of long-term opioid use for chronic non-cancer pain on misuse, abuse or addiction, overdose, **falls** and fractures](http://onlinelibrary.wiley.com/doi/10.1002/14651858.CD011062/abstract) (P) | Cochrane Back Group | N/A | 2014 |
|  | [Surgical interventions for diaphyseal fractures of the radius and ulna in children](http://onlinelibrary.wiley.com/doi/10.1002/14651858.CD007907.pub2/abstract) (R) | Cochrane Bone, Joint, and Muscle Trauma Group | 0 | 2011 |
|  | [Exercise for improving outcomes after osteoporotic vertebral fracture](http://onlinelibrary.wiley.com/doi/10.1002/14651858.CD008618.pub2/abstract) (R) | Cochrane Musculoskeletal Group | 7 | 2013 |
|  | [Rehabilitation interventions for improving physical and psychosocial functioning after hip fracture in older people](http://onlinelibrary.wiley.com/doi/10.1002/14651858.CD007624.pub3/abstract) (R) | Cochrane Bone, Joint, and Muscle Trauma Group | 9 | 2010 |
|  | [Aspiration of the elbow joint for treating radial head fractures](http://onlinelibrary.wiley.com/doi/10.1002/14651858.CD009949.pub2/abstract) (R) | Cochrane Bone, Joint, and Muscle Trauma Group | 2 | 2014 |
|  | [Physical rehabilitation for older people in long-term care](http://onlinelibrary.wiley.com/doi/10.1002/14651858.CD004294.pub3/abstract) (R) | Cochrane Stroke Group | 67 | 2013 |
|  | [Early mobilisation for elbow fractures in adults](http://onlinelibrary.wiley.com/doi/10.1002/14651858.CD008130.pub2/abstract) (R) | Cochrane Bone, Joint, and Muscle Trauma Group | 1 | 2011 |
|  | [Exercise for preventing and treating osteoporosis in postmenopausal women](http://onlinelibrary.wiley.com/doi/10.1002/14651858.CD000333.pub2/abstract) (R) | Cochrane Musculoskeletal Group | 43 | 2011 |
|  | [Surgical versus conservative interventions for displaced intra-articular calcaneal fractures](http://onlinelibrary.wiley.com/doi/10.1002/14651858.CD008628.pub2/abstract) (R) | Cochrane Bone, Joint, and Muscle Trauma Group | 4 | 2013 |
|  | [Exercise for improving balance in older people](http://onlinelibrary.wiley.com/doi/10.1002/14651858.CD004963.pub3/abstract) (R) | Cochrane Bone, Joint, and Muscle Trauma Group | 94 | 2011 |
|  | [Balance training (proprioceptive training) for patients with rheumatoid arthritis](http://onlinelibrary.wiley.com/doi/10.1002/14651858.CD007648.pub2/abstract) (R) | Cochrane Musculoskeletal Group | 0 | 2010 |
|  | [Hyperbaric oxygen therapy for the adjunctive treatment of traumatic brain injury](http://onlinelibrary.wiley.com/doi/10.1002/14651858.CD004609.pub3/abstract) (R) | Cochrane Injuries Group | 7 | 2012 |
|  | [Surgical versus conservative interventions for treating fractures of the middle third of the clavicle](http://onlinelibrary.wiley.com/doi/10.1002/14651858.CD009363.pub2/abstract) (R) | Cochrane Bone, Joint, and Muscle Trauma Group | 8 | 2013 |
|  | [Psychosocial interventions for the prevention of disability following traumatic physical injury](http://onlinelibrary.wiley.com/doi/10.1002/14651858.CD006422.pub3/abstract) (R) | Cochrane Bone, Joint, and Muscle Trauma Group | 5 | 2009 |
|  | [Percutaneous pinning for treating distal radial fractures in adults](http://onlinelibrary.wiley.com/doi/10.1002/14651858.CD006080.pub2/abstract) (R) | Cochrane Bone, Joint, and Muscle Trauma Group | 13 | 2007 |
|  | [Interventions for treating proximal humeral fractures in adults](http://onlinelibrary.wiley.com/doi/10.1002/14651858.CD000434.pub3/abstract) (R) | Cochrane Bone, Joint, and Muscle Trauma Group | 23 | 2012 |
|  | [Surgical versus non-surgical treatment for acute anterior shoulder dislocation](http://onlinelibrary.wiley.com/doi/10.1002/14651858.CD004325.pub2/abstract) (R) | Cochrane Bone, Joint, and Muscle Trauma Group | 4 | 2004 |
|  | [Surgical interventions for treating fractures of the olecranon in adults](http://onlinelibrary.wiley.com/doi/10.1002/14651858.CD010144.pub2/abstract) (R) | Cochrane Bone, Joint, and Muscle Trauma Group | 6 | 2014 |
|  | [Modest cooling therapies (35ºC to 37.5ºC) for traumatic brain injury](http://onlinelibrary.wiley.com/doi/10.1002/14651858.CD006811.pub3/abstract) (R) | Cochrane Injuries Group | 0 | 2014 |
|  | [Progressive resistance strength training for improving physical function in older adults](http://onlinelibrary.wiley.com/doi/10.1002/14651858.CD002759.pub2/abstract) (R) | Cochrane Bone, Joint, and Muscle Trauma Group | 121 | 2009 |
|  | [Rehabilitation for distal radial fractures in adults](http://onlinelibrary.wiley.com/doi/10.1002/14651858.CD003324.pub2/abstract) (R) | Cochrane Bone, Joint, and Muscle Trauma Group | 15 | 2006 |
|  | [Alendronate for the primary and secondary prevention of osteoporotic fractures in postmenopausal women](http://onlinelibrary.wiley.com/doi/10.1002/14651858.CD001155.pub2/abstract) (R) | Cochrane Musculoskeletal Group | 11 | 2008 |
|  | [Surgical interventions for treating distal humeral fractures in adults](http://onlinelibrary.wiley.com/doi/10.1002/14651858.CD009890.pub2/abstract) (R) | Cochrane Bone, Joint, and Muscle Trauma Group | 3 | 2013 |
|  | [Community screening for visual impairment in the elderly](http://onlinelibrary.wiley.com/doi/10.1002/14651858.CD001054.pub2/abstract) (R) | Cochrane Eyes and Vision Group | 5 | 2006 |
|  | [Different methods of external fixation for treating distal radial fractures in adults](http://onlinelibrary.wiley.com/doi/10.1002/14651858.CD006522.pub2/abstract) (R) | Cochrane Bone, Joint, and Muscle Trauma Group | 9 | 2008 |
|  | [Bone grafts and bone substitutes for treating distal radial fractures in adults](http://onlinelibrary.wiley.com/doi/10.1002/14651858.CD006836.pub2/abstract) (R) | Cochrane Bone, Joint, and Muscle Trauma Group | 10 | 2008 |
|  | [Etidronate for the primary and secondary prevention of osteoporotic fractures in postmenopausal women](http://onlinelibrary.wiley.com/doi/10.1002/14651858.CD003376.pub3/abstract) (R) | Cochrane Musculoskeletal Group | 11 | 2008 |
|  | [Bisphosphonate therapy for children and adolescents with secondary osteoporosis](http://onlinelibrary.wiley.com/doi/10.1002/14651858.CD005324.pub2/abstract) (R) | Cochrane Musculoskeletal Group | 6 | 2007 |
|  | [Conservative interventions for treating distal radial fractures in adults](http://onlinelibrary.wiley.com/doi/10.1002/14651858.CD000314/abstract) (R) | Cochrane Bone, Joint, and Muscle Trauma Group | 37 | 2003 |
|  | [Risedronate for the primary and secondary prevention of osteoporotic fractures in postmenopausal women](http://onlinelibrary.wiley.com/doi/10.1002/14651858.CD004523.pub3/abstract) (R) | Cochrane Musculoskeletal Group | 7 | 2008 |
|  | [Surgical interventions for anterior shoulder instability in adults](http://onlinelibrary.wiley.com/doi/10.1002/14651858.CD005077.pub2/abstract) (R) | Cochrane Bone, Joint, and Muscle Trauma Group | 3 | 2009 |
|  | [Interventions for treating traumatised permanent front teeth: luxated (dislodged) teeth](http://onlinelibrary.wiley.com/doi/10.1002/14651858.CD006203.pub2/abstract) (R) | Cochrane Oral Health Group | 0 | 2013 |
|  | [Thiazide diuretics and the risk of hip fracture](http://onlinelibrary.wiley.com/doi/10.1002/14651858.CD005185.pub2/abstract) (R) | Cochrane Hypertension Group | 21 | 2011 |
|  | [External fixation versus conservative treatment for distal radial fractures in adults](http://onlinelibrary.wiley.com/doi/10.1002/14651858.CD006194.pub2/abstract) (R) | Cochrane Bone, Joint, and Muscle Trauma Group | 15 | 2007 |
| Fire, Heat, and Hot Substances | [Interventions for promoting **smoke** alarm ownership and function](http://onlinelibrary.wiley.com/doi/10.1002/14651858.CD002246/abstract) (R) | Cochrane Injuries Group | 26 | 2001 |
|  | [Home safety education and provision of safety equipment for injury prevention](http://onlinelibrary.wiley.com/doi/10.1002/14651858.CD005014.pub3/abstract) (R) | Cochrane Injuries Group | 98 | 2012 |
|  | [Psychosocial interventions for the prevention of disability following traumatic physical injury](http://onlinelibrary.wiley.com/doi/10.1002/14651858.CD006422.pub3/abstract) (R) | Cochrane Injuries Group | 5 | 2009 |
|  | [Amniotic membrane transplantation for acute ocular **burns** (R)](http://onlinelibrary.wiley.com/doi/10.1002/14651858.CD009379.pub2/abstract) | Cochrane Eyes and Vision Group | 1 | 2012 |
|  | [Interventions for treating phosphorus **burns**](http://onlinelibrary.wiley.com/doi/10.1002/14651858.CD008805.pub3/abstract) (R) | Cochrane Wounds Group | 2 | 2014 |
|  | [Dressings for superficial and partial thickness **burns**](http://onlinelibrary.wiley.com/doi/10.1002/14651858.CD002106.pub4/abstract) (R) | Cochrane Wounds Group | 30 | 2013 |
|  | [Recombinant human growth hormone for treating **burns** and donor sites](http://onlinelibrary.wiley.com/doi/10.1002/14651858.CD008990.pub3/abstract) (R) | Cochrane Wounds Group | 13 | 2014 |
|  | [Negative pressure wound therapy for partial-thickness **burns**](http://onlinelibrary.wiley.com/doi/10.1002/14651858.CD006215.pub4/abstract) (R) | Cochrane Wounds Group | 1 | 2014 |
|  | [Antibiotic prophylaxis for preventing **burn** wound infection](http://onlinelibrary.wiley.com/doi/10.1002/14651858.CD008738.pub2/abstract) (R) | Cochrane Wounds Group | 36 | 2013 |
|  | [High-carbohydrate, high-protein, low-fat versus low-carbohydrate, high-protein, high-fat enteral feeds for **burns**](http://onlinelibrary.wiley.com/doi/10.1002/14651858.CD006122.pub3/abstract) (R) | Cochrane Injuries Group | 2 | 2012 |
|  | [Community-based interventions for the prevention of **burns** and scalds in children](http://onlinelibrary.wiley.com/doi/10.1002/14651858.CD004335.pub2/abstract) (R) | Cochrane Injuries Group | 4 | 2004 |
|  | [Topical treatment for facial **burns**](http://onlinelibrary.wiley.com/doi/10.1002/14651858.CD008058.pub2/abstract) (R) | Cochrane Wounds Group | 5 | 2013 |
|  | [Intravenous lidocaine for the treatment of background or procedural **burn** pain](http://onlinelibrary.wiley.com/doi/10.1002/14651858.CD005622.pub4/abstract) (R) | Cochrane Pain, Palliative and Supportive Care Group | 1 | 2014 |
|  | [Hyperbaric oxygen therapy for thermal **burns**](http://onlinelibrary.wiley.com/doi/10.1002/14651858.CD004727.pub2/abstract) (R) | Cochrane Injuries Group | 2 | 2004 |
|  | [Early versus delayed enteral nutrition support for **burn** injuries](http://onlinelibrary.wiley.com/doi/10.1002/14651858.CD005489.pub2/abstract) (R) | Cochrane Injuries Group | 3 | 2006 |
|  | [Immunonutrition as an adjuvant therapy for **burns**](http://onlinelibrary.wiley.com/doi/10.1002/14651858.CD007174.pub2/abstract) (R) | Cochrane Injuries Group | 16 | 2014 |
|  | [Heparin for the treatment of **burns** (R)](http://onlinelibrary.wiley.com/doi/10.1002/14651858.CD009483/abstract) | Cochrane Injuries Group | N/A | 2011 |
|  | [Early excision and grafting for **burns**](http://onlinelibrary.wiley.com/doi/10.1002/14651858.CD009715/abstract) (R) | Cochrane Wounds Group | N/A | 2012 |
|  | [Aerosolized prostacyclin for acute lung injury (ALI) and acute respiratory distress syndrome (ARDS)](http://onlinelibrary.wiley.com/doi/10.1002/14651858.CD007733.pub2/abstract) (R) | Cochrane Anaesthesia Group | 1 | 2010 |
| Poisoning | [Alkalinisation for organophosphorus pesticide **poisoning** (R)](http://onlinelibrary.wiley.com/doi/10.1002/14651858.CD004897.pub2/abstract) | Cochrane Injuries Group | 2 | 2005 |
|  | [Antidotes for acute cardenolide (cardiac glycoside) **poisoning**](http://onlinelibrary.wiley.com/doi/10.1002/14651858.CD005490.pub2/abstract) (R) | Cochrane Injuries Group | 2 | 2006 |
|  | [Urinary alkalinisation for acute chlorophenoxy herbicide **poisoning**](http://onlinelibrary.wiley.com/doi/10.1002/14651858.CD005488.pub2/abstract) (R) | Cochrane Injuries Group | 0 | 2007 |
|  | [Hyperbaric oxygen for carbon monoxide **poisoning**](http://onlinelibrary.wiley.com/doi/10.1002/14651858.CD002041.pub3/abstract) (R) | Cochrane Injuries Group | 6 | 2011 |
|  | [Oximes for acute organophosphate pesticide **poisoning**](http://onlinelibrary.wiley.com/doi/10.1002/14651858.CD005085.pub2/abstract) (R) | Cochrane Injuries Group | 3 | 2011 |
|  | [Xuebijing for paraquat **poisoning**](http://onlinelibrary.wiley.com/doi/10.1002/14651858.CD010109.pub2/abstract) (R) | Cochrane Injuries Group | 2 | 2013 |
|  | [Glucocorticoid with cyclophosphamide for paraquat-induced lung fibrosis](http://onlinelibrary.wiley.com/doi/10.1002/14651858.CD008084.pub4/abstract) (R) | Cochrane Injuries Group | 3 | 2014 |
|  | [Haemodialysis for lithium **poisoning**](http://onlinelibrary.wiley.com/doi/10.1002/14651858.CD007951/abstract) (P) | Cochrane Injuries Group | N/A | 2009 |
|  | [Home safety education and provision of safety equipment for injury prevention](http://onlinelibrary.wiley.com/doi/10.1002/14651858.CD005014.pub3/abstract) (R) | Cochrane Injuries Group | 98 | 2012 |
|  | [Household interventions for preventing domestic lead exposure in children](http://onlinelibrary.wiley.com/doi/10.1002/14651858.CD006047.pub4/abstract) (R) | Cochrane Developmental, Psychosocial and Learning Problems Group | 14 | 2014 |
|  | [Interventions for paracetamol (acetaminophen) overdose](http://onlinelibrary.wiley.com/doi/10.1002/14651858.CD003328.pub2/abstract) (R) | Cochrane Hepato-Biliary Group | 59 | 2006 |
|  | [Extracorporeal blood purification for organophosphorus pesticide **poisoning**](http://onlinelibrary.wiley.com/doi/10.1002/14651858.CD006253/abstract) (P) | Cochrane Injuries Group | N/A | 2006 |
|  | [Interventions for preventing injuries in the agricultural industry](http://onlinelibrary.wiley.com/doi/10.1002/14651858.CD006398.pub2/abstract) (R) | Cochrane Injuries Group | 5 | 2008 |
| Mechanical Forces | [Patellar tendon versus hamstring tendon autograft for anterior cruciate ligament rupture in adults](http://onlinelibrary.wiley.com/doi/10.1002/14651858.CD005960.pub2/abstract) (R) | Cochrane Bone, Joint, and Muscle Trauma Group | 19 | 2011 |
|  | [Conservative interventions for treating exercise-related musculotendinous, ligamentous and osseous groin pain](http://onlinelibrary.wiley.com/doi/10.1002/14651858.CD009565.pub2/abstract) (R) | Cochrane Bone, Joint, and Muscle Trauma Group | 2 | 2013 |
|  | [Interventions for the management of mandibular fractures](http://onlinelibrary.wiley.com/doi/10.1002/14651858.CD006087.pub3/abstract) (R) | Cochrane Oral Health Group | 12 | 2013 |
|  | [Non-legislative interventions for the promotion of cycle helmet wearing by children](http://onlinelibrary.wiley.com/doi/10.1002/14651858.CD003985.pub3/abstract) (R) | Cochrane Injuries Group | 29 | 2011 |
|  | [Conservative interventions for treating hyperextension injuries of the proximal interphalangeal joints of the fingers](http://onlinelibrary.wiley.com/doi/10.1002/14651858.CD009030.pub2/abstract) (R) | Cochrane Bone, Joint, and Muscle Trauma Group | 3 | 2013 |
|  | [Medical interventions for traumatic hyphema](http://onlinelibrary.wiley.com/doi/10.1002/14651858.CD005431.pub3/abstract) (R) | Cochrane Eyes and Vision Group | 27 | 2013 |
|  | [Antibiotic prophylaxis for mammalian **bites**](http://onlinelibrary.wiley.com/doi/10.1002/14651858.CD001738/abstract) (R) | Cochrane Wounds Group | 8 | 2001 |
|  | [Education of children and adolescents for the prevention of dog **bite** injuries](http://onlinelibrary.wiley.com/doi/10.1002/14651858.CD004726.pub2/abstract) (R) | Cochrane Injuries Group | 2 | 2009 |
|  | [Supraglottic airway devices versus tracheal intubation for airway management during general anaesthesia in obese patients](http://onlinelibrary.wiley.com/doi/10.1002/14651858.CD010105.pub2/abstract) (R) | Cochrane Anaesthesia Group | 2 | 2013 |
|  | [Preoperative fasting for preventing perioperative complications in children](http://onlinelibrary.wiley.com/doi/10.1002/14651858.CD005285.pub2/abstract) (R) | Cochrane Wounds Group | 25 | 2009 |
|  | [Aerosolized prostacyclin for acute lung injury (ALI) and acute respiratory distress syndrome (ARDS)](http://onlinelibrary.wiley.com/doi/10.1002/14651858.CD007733.pub2/abstract) (R) | Cochrane Anaesthesia Group | 1 | 2010 |
|  | [Pool fencing for preventing **drowning** of children](http://onlinelibrary.wiley.com/doi/10.1002/14651858.CD001047/abstract) (R) | Cochrane Injuries Group | 3 | 1998 |
|  | Home safety education and provision of safety equipment for injury prevention (R) | Cochrane Injuries Group | 98 | 2012 |
|  | Motivational interviewing for alcohol misuse in young adults (R) | Cochrane Drug and Alcohol Group | 66 | 2014 |
|  | Interventions for preventing injuries in problem drinkers (R) | Cochrane Injuries Group | 17 | 2004 |
| Adverse Effects of Medical Treatment | [Interventions to improve professional adherence to guidelines for prevention of device-related infections](http://onlinelibrary.wiley.com/doi/10.1002/14651858.CD006559.pub2/abstract) (R) | Cochrane Effective Practice and Organisation of Care Group | 13 | 2013 |
|  | [Anticoagulants and antiplatelet agents for preventing central venous haemodialysis catheter **malfunction** in patients with end-stage kidney disease](http://onlinelibrary.wiley.com/doi/10.1002/14651858.CD009631/abstract) (P) | Cochrane Renal Group | N/A | 2012 |
|  | [Devices for preventing percutaneous exposure injuries caused by needles in healthcare personnel](http://onlinelibrary.wiley.com/doi/10.1002/14651858.CD009740.pub2/abstract) (R) | Cochrane Occupational Safety and Health Group | 17 | 2014 |
|  | [Antibiotic and other lock treatments for tunnelled central venous catheter-related infections in children with cancer](http://onlinelibrary.wiley.com/doi/10.1002/14651858.CD008975.pub2/abstract) (R) | Cochrane Childhood Cancer Group | 3 | 2013 |
|  | [Multiple versus single lumen umbilical venous catheters for newborn infants](http://onlinelibrary.wiley.com/doi/10.1002/14651858.CD004498.pub2/abstract) (R) | Cochrane Neonatal Group | 3 | 2005 |
|  | [Prophylactic interventions after delivery of placenta for reducing bleeding during the postnatal period](http://onlinelibrary.wiley.com/doi/10.1002/14651858.CD009328.pub2/abstract) (R) | Cochrane Pregnancy and Childbirth Group | 5 | 2013 |
|  | [Surgery for stress urinary incontinence due to presumed sphincter deficiency after prostate surgery](http://onlinelibrary.wiley.com/doi/10.1002/14651858.CD008306.pub3/abstract) (R) | Cochrane Incontinence Group | 1 | 2014 |
|  | [Routine preoperative medical testing for cataract surgery](http://onlinelibrary.wiley.com/doi/10.1002/14651858.CD007293.pub3/abstract) (R) | Cochrane Eyes and Vision Group | 3 | 2012 |
| Animal Contact | [Interventions for preventing reactions to **snake** antivenom](http://onlinelibrary.wiley.com/doi/10.1002/14651858.CD002153/abstract) (R) | Cochrane Infectious Diseases Group | 2 | 1999 |
|  | [**Snake** antivenom for **snake** venom induced consumption coagulopathy](http://onlinelibrary.wiley.com/doi/10.1002/14651858.CD011428/abstract) (P) | Cochrane Injuries Group | N/A | 2014 |
|  | [Venom immunotherapy for preventing allergic reactions to insect stings](http://onlinelibrary.wiley.com/doi/10.1002/14651858.CD008838.pub2/abstract) (R) | Cochrane Skin Group | 7 | 2012 |
|  | H1-antihistamines for the treatment of anaphylaxis with and without shock (R) | Cochrane Anaesthesia Group | 0 | 2007 |
|  | [Education of children and adolescents for the prevention of **dog** bite injuries](http://onlinelibrary.wiley.com/doi/10.1002/14651858.CD004726.pub2/abstract) (R) | Cochrane Injuries Group | 2 | 2009 |
|  | [Interventions for the symptoms and signs resulting from jellyfish **stings**](http://onlinelibrary.wiley.com/doi/10.1002/14651858.CD009688.pub2/abstract) (R) | Cochrane Pain, Palliative, and Supportive Care Group | 7 | 2013 |
|  | [Antibiotic prophylaxis for mammalian bites](http://onlinelibrary.wiley.com/doi/10.1002/14651858.CD001738/abstract) (R) | Cochrane Wounds Group | 8 | 2001 |
| Self-harm | [Psychosocial and pharmacological treatments for deliberate **self harm** (R)](http://onlinelibrary.wiley.com/doi/10.1002/14651858.CD001764/abstract) | Cochrane Depression, Anxiety and Neurosis Group | 23 | 1999 |
|  | [Antidotes for acute cardenolide (cardiac glycoside) poisoning](http://onlinelibrary.wiley.com/doi/10.1002/14651858.CD005490.pub2/abstract) (R) | Cochrane Injuries Group | 2 | 2006 |
|  | [Interventions for mood and anxiety disorders, and **self harm** in young offenders](http://onlinelibrary.wiley.com/doi/10.1002/14651858.CD007195/abstract) (P) | Cochrane Depression, Anxiety, and Neurosis Group | N/A | 2008 |
|  | [Alkalinisation for organophosphorus pesticide poisoning](http://onlinelibrary.wiley.com/doi/10.1002/14651858.CD004897.pub2/abstract) (R) | Cochrane Injuries Group | 2 | 2005 |
|  | [Interventions for primary prevention of suicide in university and other post-secondary educational settings](http://onlinelibrary.wiley.com/doi/10.1002/14651858.CD009439.pub2/abstract) (R) | Cochrane Injuries Group | 8 | 2014 |
|  | [Prevention of **suicide** and suicidal behaviour in adolescents](http://onlinelibrary.wiley.com/doi/10.1002/14651858.CD007322/abstract) (P) | Cochrane Depression, Anxiety, and Neurosis Group | N/A | 2008 |
|  | [Social connectedness interventions for preventing **suicide** in young and middle-aged adults](http://onlinelibrary.wiley.com/doi/10.1002/14651858.CD009550/abstract) (P) | Cochrane Injuries Group | N/A | 2012 |
|  | [Glucocorticoid with cyclophosphamide for paraquat-induced lung fibrosis](http://onlinelibrary.wiley.com/doi/10.1002/14651858.CD008084.pub4/abstract) (R) | Cochrane Injuries Group | 3 | 2014 |
|  | [Hyperbaric oxygen for carbon monoxide poisoning](http://onlinelibrary.wiley.com/doi/10.1002/14651858.CD002041.pub3/abstract) (R) | Cochrane Injuries Group | 6 | 2011 |
| Interpersonal Violence | [Interventions for preventing injuries in problem drinkers](http://onlinelibrary.wiley.com/doi/10.1002/14651858.CD001857.pub2/abstract) (R) | Cochrane Injuries Group | 17 | 2004 |
|  | [Motivational interviewing for alcohol misuse in young adults](http://onlinelibrary.wiley.com/doi/10.1002/14651858.CD007025.pub2/abstract) (R) | Cochrane Drugs and Alcohol Group | 66 | 2014 |
|  | [Telephone delivered interventions for preventing HIV infection in HIV-negative persons](http://onlinelibrary.wiley.com/doi/10.1002/14651858.CD009190.pub2/abstract) (R) | Cochrane HIV/AIDS Group | 1 | 2013 |
|  | [Interventions in the alcohol server setting for preventing injuries](http://onlinelibrary.wiley.com/doi/10.1002/14651858.CD005244.pub3/abstract) (R) | Cochrane Injuries Group | 23 | 2008 |
|  | [Domestic violence screening and intervention programmes for adults with dental or facial injury](http://onlinelibrary.wiley.com/doi/10.1002/14651858.CD004486.pub3/abstract) (R) | Cochrane Oral Health Group | 0 | 2010 |
|  | [Cognitive behavioural therapy for men who physically abuse their female partner](http://onlinelibrary.wiley.com/doi/10.1002/14651858.CD006048.pub2/abstract) (R) | Cochrane Developmental, Psychosocial and Learning Problems Group | 6 | 2007 |
|  | [Family and parenting interventions in children and adolescents with conduct disorder and delinquency aged 10-17](http://onlinelibrary.wiley.com/doi/10.1002/14651858.CD003015/abstract) (R) | Cochrane Developmental, Psychosocial and Learning Problems Group | 8 | 2001 |
|  | [Educational and skills-based interventions for preventing relationship and dating violence in adolescents and young adults](http://onlinelibrary.wiley.com/doi/10.1002/14651858.CD004534.pub3/abstract) (R) | Cochrane Developmental, Psychosocial and Learning Problems Group | 38 | 2013 |
|  | [School-based education programmes for the prevention of child sexual abuse](http://onlinelibrary.wiley.com/doi/10.1002/14651858.CD004380.pub2/abstract) (R) | Cochrane Developmental, Psychosocial and Learning Problems Group | 15 | 2007 |
|  | [Effectiveness and safety of HIV post-exposure prophylaxis after sexual, injecting-drug-use or other non-occupational exposure](http://onlinelibrary.wiley.com/doi/10.1002/14651858.CD005273/abstract) (P) | Cochrane HIV/AIDS Group | N/A | 2005 |
|  | [Interventions for treating traumatised permanent front teeth: luxated (dislodged) teeth](http://onlinelibrary.wiley.com/doi/10.1002/14651858.CD006203.pub2/abstract) (R) | Cochrane Oral Health Group | 0 | 2013 |
|  | [Advocacy interventions to reduce or eliminate violence and promote the physical and psychosocial well-being of women who experience intimate partner abuse](http://onlinelibrary.wiley.com/doi/10.1002/14651858.CD005043.pub2/abstract) (R) | Cochrane Developmental, Psychosocial and Learning Problems Group | 10 | 2009 |
|  | [Combined pharmacotherapy and psychological therapies for post traumatic stress disorder (PTSD)](http://onlinelibrary.wiley.com/doi/10.1002/14651858.CD007316.pub2/abstract) (R) | Cochrane Depression, Anxiety and Neurosis Group | 4 | 2010 |
|  | [Pharmacological interventions for preventing post-traumatic stress disorder (PTSD)](http://onlinelibrary.wiley.com/doi/10.1002/14651858.CD006239.pub2/abstract) (R) | Cochrane Depression, Anxiety and Neurosis Group | 9 | 2014 |
|  | [Psychoanalytic/psychodynamic psychotherapy for children and adolescents who have been sexually abused](http://onlinelibrary.wiley.com/doi/10.1002/14651858.CD008162.pub2/abstract) (R) | Cochrane Developmental, Psychosocial and Learning Problems Group | 0 | 2013 |
|  | [Telephone delivered interventions for preventing HIV infection in HIV-negative persons](http://onlinelibrary.wiley.com/doi/10.1002/14651858.CD009190.pub2/abstract) (R) | Cochrane HIV/AIDS Group | 1 | 2013 |
|  | [Pharmacological interventions for those who have sexually offended or are at risk of offending](http://onlinelibrary.wiley.com/doi/10.1002/14651858.CD007989.pub2/abstract) (R) | Cochrane Developmental, Psychosocial and Learning Problems Group | 7 | 2015 |
|  | [Psychological therapies for the treatment of post-traumatic stress disorder in children and adolescents](http://onlinelibrary.wiley.com/doi/10.1002/14651858.CD006726.pub2/abstract) (R) | Cochrane Depression, Anxiety and Neurosis Group | 14 | 2012 |
|  | [Psychological interventions for adults who have sexually offended or are at risk of offending](http://onlinelibrary.wiley.com/doi/10.1002/14651858.CD007507.pub2/abstract) (R) | Cochrane Developmental, Psychosocial and Learning Problems Group | 10 | 2012 |
|  | [Psychosocial interventions for adults who were sexually abused as children](http://onlinelibrary.wiley.com/doi/10.1002/14651858.CD010099/abstract) (R) | Cochrane Developmental, Psychosocial and Learning Problems Group | N/A | 2012 |
|  | [Individual and group-based parenting programmes for the treatment of physical child abuse and neglect](http://onlinelibrary.wiley.com/doi/10.1002/14651858.CD005463.pub2/abstract) (R) | Cochrane Developmental, Psychosocial and Learning Problems Group | 7 | 2006 |
|  | [Kinship care for the safety, permanency, and well-being of children removed from the home for **maltreatment**](http://onlinelibrary.wiley.com/doi/10.1002/14651858.CD006546.pub3/abstract) (R) | Cochrane Developmental, Psychosocial and Learning Problems Group | 102 | 2014 |
|  | [Child-focused cognitive behavioural therapy for children who have been physically abused](http://onlinelibrary.wiley.com/doi/10.1002/14651858.CD007838/abstract) (P) | Cochrane Developmental, Psychosocial and Learning Problems Group | N/A | 2009 |
|  | [Cognitive-behavioural therapy for parents who have physically abused their children](http://onlinelibrary.wiley.com/doi/10.1002/14651858.CD007329/abstract) (P) | Cochrane Developmental, Psychosocial and Learning Problems Group | N/A | 2008 |
|  | [Psychological interventions to prevent recurrence of emotional abuse of children by their parents](http://onlinelibrary.wiley.com/doi/10.1002/14651858.CD010725/abstract) (R) | Cochrane Developmental, Psychosocial and Learning Problems Group | N/A | 2013 |
| Exposure to Forces of Nature | Electric fans for reducing adverse health impacts in heatwaves (R) | Cochrane Gynaecological Cancer Group | 0 | 2012 |
| Collective Violence and Legal Intervention |  |  |  |  |
