# Supplementary material for: Identifying gaps for research prioritisation: Global burden of external causes of injury as reflected in the Cochrane Database of Systematic Reviews
Source: Injury. 2016 May;47(5):1151–7. doi: 10.1016/j.injury.2015.12.019 (PMC4862959; doi:10.1016/j.injury.2015.12.019)
Supplement: Supplementary file 2 [file mmc2.docx]

**eTable 2: Excluded titles generated from search of 12 injury and trauma conditions studied by GBD 2010 in *The Cochrane Database of Systematic Reviews***

| **Condition** | **Review (R) or Protocol (P)** | **Excluded without Discrepancy** | **Excluded after Consensus Review** |
| --- | --- | --- | --- |
| Road Injury | Interventions to reduce risky sexual behaviour for preventing HIV infection in workers in occupational settings (R) | X |  |
|  | [Diagnostic tests for Autism Spectrum Disorders (ASD) in preschool children](http://onlinelibrary.wiley.com/doi/10.1002/14651858.CD009044/abstract) (P) | X |  |
|  | [Legislative smoking bans for reducing secondhand smoke exposure, smoking prevalence and tobacco consumption](http://onlinelibrary.wiley.com/doi/10.1002/14651858.CD005992.pub2/abstract) (R) | X |  |
|  | [Mobile phone messaging for preventive health care](http://onlinelibrary.wiley.com/doi/10.1002/14651858.CD007457.pub2/abstract) (R) | X |  |
|  | [Later school start times for supporting the education, health and well-being of high school students](http://onlinelibrary.wiley.com/doi/10.1002/14651858.CD009467/abstract) (P) | X |  |
|  | [Interventions to encourage uptake of cancer screening for people with severe mental illness](http://onlinelibrary.wiley.com/doi/10.1002/14651858.CD009641.pub2/abstract) (R) | X |  |
|  | [Social norms information for alcohol misuse in university and college students](http://onlinelibrary.wiley.com/doi/10.1002/14651858.CD006748.pub3/abstract) (R) | X |  |
|  | [Motivational interviewing for alcohol misuse in young adults](http://onlinelibrary.wiley.com/doi/10.1002/14651858.CD007025.pub2/abstract) (R) | X |  |
|  | [High-dose chemotherapy and autologous haematopoietic stem cell rescue for children with high-risk neuroblastoma](http://onlinelibrary.wiley.com/doi/10.1002/14651858.CD006301.pub3/abstract) (R) | X |  |
|  | [Exercise interventions on health-related quality of life for people with cancer during active treatment](http://onlinelibrary.wiley.com/doi/10.1002/14651858.CD008465.pub2/abstract) (R) | X |  |
|  | [Optimal intensity and type of leg exercise training for people with chronic obstructive pulmonary disease](http://onlinelibrary.wiley.com/doi/10.1002/14651858.CD008008.pub2/abstract) (R) | X |  |
|  | [Thyroid hormones for preventing neurodevelopmental impairment in preterm infants](http://onlinelibrary.wiley.com/doi/10.1002/14651858.CD001070/abstract) (R) | X |  |
|  | [Prophylactic postnatal thyroid hormones for prevention of morbidity and mortality in preterm infants](http://onlinelibrary.wiley.com/doi/10.1002/14651858.CD005948.pub2/abstract) (R) | X |  |
|  | [Interventions for reducing the risk of mother-to-child transmission of HIV infection](http://onlinelibrary.wiley.com/doi/10.1002/14651858.CD000102/abstract) (R) | X |  |
|  | [Topical pimecrolimus for eczema](http://onlinelibrary.wiley.com/doi/10.1002/14651858.CD005500.pub2/abstract) (R) | X |  |
|  | [Snoezelen for dementia](http://onlinelibrary.wiley.com/doi/10.1002/14651858.CD003152/abstract) (R) | X |  |
|  | [Ketanserin for Raynaud's phenomenon in progressive systemic sclerosis](http://onlinelibrary.wiley.com/doi/10.1002/14651858.CD000954/abstract) (R) | X |  |
|  | [Exercise for treating fibromyalgia syndrome](http://onlinelibrary.wiley.com/doi/10.1002/14651858.CD003786.pub2/abstract) (R) | X |  |
|  | [Occupational therapy for rheumatoid arthritis](http://onlinelibrary.wiley.com/doi/10.1002/14651858.CD003114.pub2/abstract) (R) | X |  |
|  | [Occupational therapy for multiple sclerosis](http://onlinelibrary.wiley.com/doi/10.1002/14651858.CD003608/abstract) (R) | X |  |
|  | [Dynamic exercise programs (aerobic capacity and/or muscle strength training) in patients with rheumatoid arthritis](http://onlinelibrary.wiley.com/doi/10.1002/14651858.CD006853.pub2/abstract) (R) | X |  |
|  | [Treatment of acute cryptococcal meningitis in HIV infected adults, with an emphasis on resource-limited settings](http://onlinelibrary.wiley.com/doi/10.1002/14651858.CD005647.pub2/abstract) (R) | X |  |
|  | [Psychosocial interventions for erectile dysfunction](http://onlinelibrary.wiley.com/doi/10.1002/14651858.CD004825.pub2/abstract) (R) | X |  |
|  | [Partial exchange transfusion to prevent neurodevelopmental disability in infants with polycythemia](http://onlinelibrary.wiley.com/doi/10.1002/14651858.CD005089.pub2/abstract) (R) | X |  |
|  | [Interventions for erosive lichen planus affecting mucosal sites](http://onlinelibrary.wiley.com/doi/10.1002/14651858.CD008092.pub2/abstract) (R) | X |  |
|  | [Non-fluoride topical remineralising agents containing calcium and/or phosphate for controlling dental caries](http://onlinelibrary.wiley.com/doi/10.1002/14651858.CD009732/abstract) (R) | X |  |
|  | [Interventions for actinic keratoses](http://onlinelibrary.wiley.com/doi/10.1002/14651858.CD004415.pub2/abstract) (R) | X |  |
|  | [Interventions for treating scabies](http://onlinelibrary.wiley.com/doi/10.1002/14651858.CD000320.pub2/abstract) (R) | X |  |
|  | [Dressings and topical agents for arterial leg ulcers](http://onlinelibrary.wiley.com/doi/10.1002/14651858.CD001836.pub2/abstract) (R) | X |  |
|  | [Psychological therapies for the treatment of post-traumatic stress disorder in children and adolescents](http://onlinelibrary.wiley.com/doi/10.1002/14651858.CD006726.pub2/abstract) (R) | X |  |
|  | [Dehydroepiandrosterone (DHEA) supplementation for cognitive function in healthy elderly people](http://onlinelibrary.wiley.com/doi/10.1002/14651858.CD006221/abstract) (R) | X |  |
|  | [Nasal versus oral route for placing feeding tubes in preterm or low birth weight infants](http://onlinelibrary.wiley.com/doi/10.1002/14651858.CD003952.pub3/abstract) (R) | X |  |
|  | [Interventions for drooling in children with cerebral palsy](http://onlinelibrary.wiley.com/doi/10.1002/14651858.CD008624.pub3/abstract) (R) | X |  |
|  | [Dopamine transporter imaging for the diagnosis of dementia with Lewy bodies](http://onlinelibrary.wiley.com/doi/10.1002/14651858.CD010633.pub2/abstract) (R) | X |  |
|  | [Pharmacologic interventions for treating phantom limb pain](http://onlinelibrary.wiley.com/doi/10.1002/14651858.CD006380.pub2/abstract) (R) | X |  |
|  | [^18^F-FDG PET for the early diagnosis of Alzheimer’s disease dementia and other dementias in people with mild cognitive impairment (MCI)](http://onlinelibrary.wiley.com/doi/10.1002/14651858.CD010632.pub2/abstract) (R) | X |  |
|  | [Centre-based day care for children younger than five years of age in low- and middle-income countries](http://onlinelibrary.wiley.com/doi/10.1002/14651858.CD010543.pub2/abstract) (R) | X |  |
|  | [Intratracheal instillation of corticosteroids using surfactant as a **vehicle** for the prevention of chronic lung disease in preterm infants with respiratory distress syndrome](http://onlinelibrary.wiley.com/doi/10.1002/14651858.CD009064/abstract) (P) | X |  |
|  | [Intramedullary nailing for tibial shaft fractures in adults](http://onlinelibrary.wiley.com/doi/10.1002/14651858.CD008241.pub2/abstract) (R) | X |  |
|  | [Pool fencing for preventing drowning of children](http://onlinelibrary.wiley.com/doi/10.1002/14651858.CD001047/abstract) (R) | X |  |
|  | [Spermicide used alone for contraception](http://onlinelibrary.wiley.com/doi/10.1002/14651858.CD005218.pub4/abstract) (R) | X |  |
|  | [Interventions for cutaneous molluscum contagiosum](http://onlinelibrary.wiley.com/doi/10.1002/14651858.CD004767.pub3/abstract) (R) | X |  |
|  | [Continuous positive airway pressure delivery interfaces for obstructive sleep apnoea](http://onlinelibrary.wiley.com/doi/10.1002/14651858.CD005308.pub2/abstract) (R) | X |  |
|  | [Interventions in the alcohol server setting for preventing injuries](http://onlinelibrary.wiley.com/doi/10.1002/14651858.CD005244.pub3/abstract) (R) | X |  |
|  | [Fluoridated milk for preventing dental caries](http://onlinelibrary.wiley.com/doi/10.1002/14651858.CD003876.pub2/abstract) (R) | X |  |
|  | [Antibiotics versus placebo for acute bacterial conjunctivitis](http://onlinelibrary.wiley.com/doi/10.1002/14651858.CD001211.pub3/abstract) (R) | X |  |
|  | [Antibiotics and antiseptics for venous leg ulcers](http://onlinelibrary.wiley.com/doi/10.1002/14651858.CD003557.pub5/abstract) (R) | X |  |
|  | [Interventions for preventing occupational irritant hand dermatitis](http://onlinelibrary.wiley.com/doi/10.1002/14651858.CD004414.pub2/abstract) (R) | X |  |
|  | [Smartphone and tablet self management apps for asthma](http://onlinelibrary.wiley.com/doi/10.1002/14651858.CD010013.pub2/abstract) (R) | X |  |
|  | [Pharmacological interventions for preventing post-traumatic stress disorder (PTSD)](http://onlinelibrary.wiley.com/doi/10.1002/14651858.CD006239.pub2/abstract) (R) | X |  |
|  | [Interventions for promoting reintegration and reducing harmful behaviour and lifestyles in street-connected children and young people](http://onlinelibrary.wiley.com/doi/10.1002/14651858.CD009823.pub2/abstract) (R) | X |  |
| Other Transport Injury | Corticosteroids for parasitic eosinophilic meningitis (R) | X |  |
|  | [**Helicopter** emergency medical services for adults with major trauma](http://onlinelibrary.wiley.com/doi/10.1002/14651858.CD009228.pub2/abstract) (R) | X |  |
|  | Specialty teams for neonatal transport to neonatal intensive care units for prevention of morbidity and mortality (R) | X |  |
| Falls | [Effect of longer-term modest salt reduction on blood pressure](http://onlinelibrary.wiley.com/doi/10.1002/14651858.CD004937.pub2/abstract) (R) | X |  |
|  | [Mast-cell stabilising agents to prevent exercise-induced bronchoconstriction](http://onlinelibrary.wiley.com/doi/10.1002/14651858.CD002307/abstract) (R) | X |  |
|  | [Oxygen therapy for cystic fibrosis](http://onlinelibrary.wiley.com/doi/10.1002/14651858.CD003884.pub4/abstract) (R) | X |  |
|  | [Monosodium glutamate avoidance for chronic asthma in adults and children](http://onlinelibrary.wiley.com/doi/10.1002/14651858.CD004357.pub4/abstract) (R) | X |  |
|  | [Nedocromil sodium for preventing exercise-induced bronchoconstriction](http://onlinelibrary.wiley.com/doi/10.1002/14651858.CD001183/abstract) (R) | X |  |
|  | [Interventions for preventing delirium in older people in institutional long-term care](http://onlinelibrary.wiley.com/doi/10.1002/14651858.CD009537.pub2/abstract) (R) | X |  |
|  | [Inhaled corticosteroids compared to placebo for prevention of exercise induced bronchoconstriction](http://onlinelibrary.wiley.com/doi/10.1002/14651858.CD002739.pub3/abstract) (R) | X |  |
|  | [Physiotherapy versus placebo or no intervention in Parkinson's disease](http://onlinelibrary.wiley.com/doi/10.1002/14651858.CD002817.pub4/abstract) (R) | X |  |
|  | [Carbonic anhydrase inhibitors for hypercapnic ventilatory failure in chronic obstructive pulmonary disease](http://onlinelibrary.wiley.com/doi/10.1002/14651858.CD002881/abstract) (R) | X |  |
|  | [Progesterone for premenstrual syndrome](http://onlinelibrary.wiley.com/doi/10.1002/14651858.CD003415.pub4/abstract) (R) | X |  |
|  | [Dietary advice for reducing cardiovascular risk](http://onlinelibrary.wiley.com/doi/10.1002/14651858.CD002128.pub5/abstract) (R) | X |  |
|  | [Incentive spirometry for prevention of postoperative pulmonary complications in upper abdominal surgery](http://onlinelibrary.wiley.com/doi/10.1002/14651858.CD006058.pub3/abstract) (R) | X |  |
|  | [Red cell transfusion management for patients undergoing cardiac surgery for congenital heart disease](http://onlinelibrary.wiley.com/doi/10.1002/14651858.CD009752.pub2/abstract) (R) | X |  |
|  | [Oxcarbazepine for acute affective episodes in bipolar disorder](http://onlinelibrary.wiley.com/doi/10.1002/14651858.CD004857.pub2/abstract) (R) | X |  |
|  | [Heparin versus 0.9% sodium chloride intermittent flushing for prevention of occlusion in central venous catheters in adults](http://onlinelibrary.wiley.com/doi/10.1002/14651858.CD008462.pub2/abstract) (R) | X |  |
|  | [Pharmacotherapies for sleep disturbances in Alzheimer's disease](http://onlinelibrary.wiley.com/doi/10.1002/14651858.CD009178.pub2/abstract) (R) | X |  |
|  | [Smartphone and tablet self management apps for asthma](http://onlinelibrary.wiley.com/doi/10.1002/14651858.CD010013.pub2/abstract) (R) | X |  |
|  | [Long-acting beta_2_-agonist in addition to tiotropium versus either tiotropium or long-acting beta_2_-agonist alone for chronic obstructive pulmonary disease](http://onlinelibrary.wiley.com/doi/10.1002/14651858.CD008989.pub2/abstract) (R) | X |  |
|  | [Cognitive rehabilitation for spatial neglect following stroke](http://onlinelibrary.wiley.com/doi/10.1002/14651858.CD003586.pub3/abstract) (R) | X |  |
|  | [Correction of chronic metabolic acidosis for chronic kidney disease patients](http://onlinelibrary.wiley.com/doi/10.1002/14651858.CD001890.pub3/abstract) (R) | X |  |
|  | [Ayurvedic medicine for schizophrenia](http://onlinelibrary.wiley.com/doi/10.1002/14651858.CD006867/abstract) (R) | X |  |
|  | [Prosthetic rehabilitation for older dysvascular people following a unilateral transfemoral amputation](http://onlinelibrary.wiley.com/doi/10.1002/14651858.CD005260.pub3/abstract) (R) | X |  |
|  | [Cholinesterase inhibitors for dementia with Lewy bodies](http://onlinelibrary.wiley.com/doi/10.1002/14651858.CD003672/abstract) (R) | X |  |
|  | [Beta_2_-agonists for exercise-induced asthma](http://onlinelibrary.wiley.com/doi/10.1002/14651858.CD003564.pub3/abstract) (R) | X |  |
|  | [Valproate preparations for agitation in dementia](http://onlinelibrary.wiley.com/doi/10.1002/14651858.CD003945.pub3/abstract) (R) | X |  |
|  | [Low versus high haemoglobin concentration threshold for blood transfusion for preventing morbidity and mortality in very low birth weight infants](http://onlinelibrary.wiley.com/doi/10.1002/14651858.CD000512.pub2/abstract) (R) | X |  |
|  | [Vaccines for preventing smallpox](http://onlinelibrary.wiley.com/doi/10.1002/14651858.CD004913.pub2/abstract) (R) | X |  |
|  | [Drugs for treatment of very high blood pressure during pregnancy](http://onlinelibrary.wiley.com/doi/10.1002/14651858.CD001449.pub3/abstract) (R) | X |  |
|  | [Cholinesterase inhibitors for dementia with Lewy bodies, Parkinson's disease dementia and cognitive impairment in Parkinson's disease](http://onlinelibrary.wiley.com/doi/10.1002/14651858.CD006504.pub2/abstract) (R) | X |  |
|  | [Circuit class therapy for improving mobility after stroke](http://onlinelibrary.wiley.com/doi/10.1002/14651858.CD007513.pub2/abstract) (R) | X |  |
|  | [Rubber band ligation versus excisional haemorrhoidectomy for haemorrhoids](http://onlinelibrary.wiley.com/doi/10.1002/14651858.CD005034.pub2/abstract) (R) | X |  |
|  | [Treadmill interventions with partial body weight support in children under six years of age at risk of neuromotor delay](http://onlinelibrary.wiley.com/doi/10.1002/14651858.CD009242.pub2/abstract) (R) | X |  |
|  | [Home safety education and provision of safety equipment for injury prevention](http://onlinelibrary.wiley.com/doi/10.1002/14651858.CD005014.pub3/abstract) (R) | X |  |
|  | [Antihypertensive drug therapy for mild to moderate hypertension during pregnancy](http://onlinelibrary.wiley.com/doi/10.1002/14651858.CD002252.pub3/abstract) (R) | X |  |
|  | [Imipramine for neuropathic pain in adults](http://onlinelibrary.wiley.com/doi/10.1002/14651858.CD010769.pub2/abstract) (R) | X |  |
|  | [Doxapram for ventilatory failure due to exacerbations of chronic obstructive pulmonary disease](http://onlinelibrary.wiley.com/doi/10.1002/14651858.CD000223/abstract) (R) | X |  |
|  | [Interventions for treating depression after stroke](http://onlinelibrary.wiley.com/doi/10.1002/14651858.CD003437.pub3/abstract) (R) | X |  |
|  | [Prophylactic vitamin K for vitamin K deficiency bleeding in neonates](http://onlinelibrary.wiley.com/doi/10.1002/14651858.CD002776/abstract) (R) | X |  |
|  | [Desipramine for neuropathic pain in adults](http://onlinelibrary.wiley.com/doi/10.1002/14651858.CD011003.pub2/abstract) (R) | X |  |
|  | [Interventions for preventing and reducing the use of physical restraints in long-term geriatric care](http://onlinelibrary.wiley.com/doi/10.1002/14651858.CD007546.pub2/abstract) (R) | X |  |
|  | [Anticholinergic therapy for chronic asthma in children over two years of age](http://onlinelibrary.wiley.com/doi/10.1002/14651858.CD003535/abstract) (R) | X |  |
|  | [Early versus late erythropoietin for preventing red blood cell transfusion in preterm and/or low birth weight infants](http://onlinelibrary.wiley.com/doi/10.1002/14651858.CD004865.pub3/abstract) (R) | X |  |
|  | [Altered dietary salt intake for preventing and treating diabetic kidney disease](http://onlinelibrary.wiley.com/doi/10.1002/14651858.CD006763.pub2/abstract) (R) | X |  |
|  | [Pulse oximetry for perioperative monitoring](http://onlinelibrary.wiley.com/doi/10.1002/14651858.CD002013.pub3/abstract) (R) | X |  |
|  | [Late erythropoietin for preventing red blood cell transfusion in preterm and/or low birth weight infants](http://onlinelibrary.wiley.com/doi/10.1002/14651858.CD004868.pub4/abstract) (R) | X |  |
|  | [Face-to-face interventions for promoting physical activity](http://onlinelibrary.wiley.com/doi/10.1002/14651858.CD010392.pub2/abstract) (R) | X |  |
|  | [Parent training interventions for Attention Deficit Hyperactivity Disorder (ADHD) in children aged 5 to 18 years](http://onlinelibrary.wiley.com/doi/10.1002/14651858.CD003018.pub3/abstract) (R) | X |  |
|  | [Carbamazepine for chronic neuropathic pain and fibromyalgia in adults](http://onlinelibrary.wiley.com/doi/10.1002/14651858.CD005451.pub3/abstract) (R) | X |  |
|  | [Interventions for preventing mastitis after childbirth](http://onlinelibrary.wiley.com/doi/10.1002/14651858.CD007239.pub3/abstract) (R) | X |  |
|  | [Sacral nerve stimulation for faecal incontinence and constipation in adults](http://onlinelibrary.wiley.com/doi/10.1002/14651858.CD004464.pub2/abstract) (R) | X |  |
|  | [Acellular vaccines for preventing whooping cough in children](http://onlinelibrary.wiley.com/doi/10.1002/14651858.CD001478.pub6/abstract) (R) | X |  |
|  | [Ionisers for chronic asthma](http://onlinelibrary.wiley.com/doi/10.1002/14651858.CD002986.pub2/abstract) (R) | X |  |
|  | [Femoral nerve blocks for acute postoperative pain after knee replacement surgery](http://onlinelibrary.wiley.com/doi/10.1002/14651858.CD009941.pub2/abstract) (R) | X |  |
|  | [Interventions at caesarean section for reducing the risk of aspiration pneumonitis](http://onlinelibrary.wiley.com/doi/10.1002/14651858.CD004943.pub4/abstract) (R) | X |  |
|  | [Artemisinin-based combination therapy for treating uncomplicated malaria](http://onlinelibrary.wiley.com/doi/10.1002/14651858.CD007483.pub2/abstract) (R) | X |  |
|  | [Interventions for primary (intrinsic) tracheomalacia in children](http://onlinelibrary.wiley.com/doi/10.1002/14651858.CD005304.pub3/abstract) (R) | X |  |
|  | [Beta-blocker therapy for tremor in Parkinson's disease](http://onlinelibrary.wiley.com/doi/10.1002/14651858.CD003361/abstract) (R) | X |  |
|  | [Nitrates for achalasia](http://onlinelibrary.wiley.com/doi/10.1002/14651858.CD002299.pub2/abstract) (R) | X |  |
|  | [Gabapentin for chronic neuropathic pain and fibromyalgia in adults](http://onlinelibrary.wiley.com/doi/10.1002/14651858.CD007938.pub3/abstract) (R) | X |  |
|  | [A therapeutic-only versus prophylactic platelet transfusion strategy for preventing bleeding in patients with haematological disorders after chemotherapy or stem cell transplantation](http://onlinelibrary.wiley.com/doi/10.1002/14651858.CD010981/abstract) (R) | X |  |
|  | [Lamotrigine for chronic neuropathic pain and fibromyalgia in adults](http://onlinelibrary.wiley.com/doi/10.1002/14651858.CD006044.pub4/abstract) (R) | X |  |
|  | [Interventions for tobacco use prevention in Indigenous youth](http://onlinelibrary.wiley.com/doi/10.1002/14651858.CD009325.pub2/abstract) (R) | X |  |
|  | [Melatonin for the prevention and treatment of jet lag](http://onlinelibrary.wiley.com/doi/10.1002/14651858.CD001520/abstract) (R) | X |  |
|  | [Interventions for preventing lower limb soft-tissue running injuries](http://onlinelibrary.wiley.com/doi/10.1002/14651858.CD001256.pub2/abstract) (R) | X |  |
|  | [Helmets for preventing head and facial injuries in bicyclists](http://onlinelibrary.wiley.com/doi/10.1002/14651858.CD001855/abstract) (R) | X |  |
|  | [Psychosocial interventions for reducing antipsychotic medication in care home residents](http://onlinelibrary.wiley.com/doi/10.1002/14651858.CD008634.pub2/abstract) (R) | X |  |
|  | [Interventions based on the Theory of Mind cognitive model for autism spectrum disorder (ASD)](http://onlinelibrary.wiley.com/doi/10.1002/14651858.CD008785.pub2/abstract) (R) | X |  |
|  | [Barbiturates for acute traumatic brain injury](http://onlinelibrary.wiley.com/doi/10.1002/14651858.CD000033.pub2/abstract) (R) | X |  |
|  | [Time course for blood pressure lowering of dihydropyridine calcium channel blockers](http://onlinelibrary.wiley.com/doi/10.1002/14651858.CD010052.pub2/abstract) (R) | X |  |
|  | [Perioperative beta-blockers for preventing surgery-related mortality and morbidity](http://onlinelibrary.wiley.com/doi/10.1002/14651858.CD004476.pub2/abstract) (R) | X |  |
|  | [Zonisamide for neuropathic pain in adults](http://onlinelibrary.wiley.com/doi/10.1002/14651858.CD011241.pub2/abstract) (R) | X |  |
|  | [Tai chi for primary prevention of cardiovascular disease](http://onlinelibrary.wiley.com/doi/10.1002/14651858.CD010366.pub2/abstract) (R) | X |  |
|  | [Mucolytic agents for chronic bronchitis or chronic obstructive pulmonary disease](http://onlinelibrary.wiley.com/doi/10.1002/14651858.CD001287.pub4/abstract) (R) | X |  |
|  | [Antiepileptic drugs for neuropathic pain and fibromyalgia - an overview of Cochrane reviews](http://onlinelibrary.wiley.com/doi/10.1002/14651858.CD010567.pub2/abstract) (R) | X |  |
|  | [Early postnatal discharge from hospital for healthy mothers and term infants](http://onlinelibrary.wiley.com/doi/10.1002/14651858.CD002958/abstract) (R) | X |  |
|  | [Physical exercise for sleep problems in adults aged 60+](http://onlinelibrary.wiley.com/doi/10.1002/14651858.CD003404/abstract) (R) | X |  |
|  | [Nedocromil sodium versus sodium cromoglycate for preventing exercise-induced bronchoconstriction](http://onlinelibrary.wiley.com/doi/10.1002/14651858.CD002731/abstract) (R) | X |  |
|  | [Heliox for treatment of exacerbations of chronic obstructive pulmonary disease](http://onlinelibrary.wiley.com/doi/10.1002/14651858.CD003571/abstract) (R) | X |  |
|  | [Topiramate for neuropathic pain and fibromyalgia in adults](http://onlinelibrary.wiley.com/doi/10.1002/14651858.CD008314.pub3/abstract) (R) | X |  |
|  | [Psychological interventions for adults who have sexually offended or are at risk of offending](http://onlinelibrary.wiley.com/doi/10.1002/14651858.CD007507.pub2/abstract) (R) | X |  |
|  | [Antimicrobial drugs for treating cholera](http://onlinelibrary.wiley.com/doi/10.1002/14651858.CD008625.pub2/abstract) (R) | X |  |
|  | [Maternal position during caesarean section for preventing maternal and neonatal complications](http://onlinelibrary.wiley.com/doi/10.1002/14651858.CD007623.pub3/abstract) (R) | X |  |
|  | [Remote and web 2.0 interventions for promoting physical activity](http://onlinelibrary.wiley.com/doi/10.1002/14651858.CD010395.pub2/abstract) (R) | X |  |
|  | [Intrapartum antibiotics for known maternal Group B streptococcal colonization](http://onlinelibrary.wiley.com/doi/10.1002/14651858.CD007467.pub4/abstract) (R) | X |  |
|  | [Hyperventilation therapy for acute traumatic brain injury](http://onlinelibrary.wiley.com/doi/10.1002/14651858.CD000566/abstract) (R) | X |  |
|  | [Effects of sevoflurane versus other general anaesthesia on emergence agitation in children](http://onlinelibrary.wiley.com/doi/10.1002/14651858.CD007084.pub2/abstract) (R) | X |  |
|  | [Oxycodone for neuropathic pain and fibromyalgia in adults](http://onlinelibrary.wiley.com/doi/10.1002/14651858.CD010692.pub2/abstract) (R) | X |  |
|  | [Levetiracetam for neuropathic pain in adults](http://onlinelibrary.wiley.com/doi/10.1002/14651858.CD010943.pub2/abstract) (R) | X |  |
|  | [Non-pharmaceutical management of respiratory morbidity in children with severe global developmental delay](http://onlinelibrary.wiley.com/doi/10.1002/14651858.CD010382.pub2/abstract) (R) | X |  |
|  | [Antibiotic treatment for *Burkholderia cepacia* complex in people with cystic fibrosis experiencing a pulmonary exacerbation](http://onlinelibrary.wiley.com/doi/10.1002/14651858.CD009529.pub2/abstract) (R) | X |  |
|  | [Honey for acute cough in children](http://onlinelibrary.wiley.com/doi/10.1002/14651858.CD007094.pub4/abstract) (R) | X |  |
|  | [Surgical techniques for the removal of mandibular wisdom teeth](http://onlinelibrary.wiley.com/doi/10.1002/14651858.CD004345.pub2/abstract) (R) | X |  |
|  | [Routine or selective carotid artery shunting for carotid endarterectomy (and different methods of monitoring in selective shunting)](http://onlinelibrary.wiley.com/doi/10.1002/14651858.CD000190.pub3/abstract) (R) | X |  |
|  | [Garlic for the prevention of cardiovascular morbidity and mortality in hypertensive patients](http://onlinelibrary.wiley.com/doi/10.1002/14651858.CD007653.pub2/abstract) (R) | X |  |
|  | [Oxygen therapy in the pre-hospital setting for acute exacerbations of chronic obstructive pulmonary disease](http://onlinelibrary.wiley.com/doi/10.1002/14651858.CD005534.pub2/abstract) (R) | X |  |
|  | [Interventions for age-related visual problems in patients with stroke](http://onlinelibrary.wiley.com/doi/10.1002/14651858.CD008390.pub2/abstract) (R) | X |  |
|  | [Atrial natriuretic peptide for preventing and treating acute kidney injury](http://onlinelibrary.wiley.com/doi/10.1002/14651858.CD006028.pub2/abstract) (R) | X |  |
|  | [Pharmacological interventions for sleepiness and sleep disturbances caused by shift work](http://onlinelibrary.wiley.com/doi/10.1002/14651858.CD009776.pub2/abstract) (R) | X |  |
|  | [Intravenous immunoglobulin as adjuvant therapy for Wegener's granulomatosis](http://onlinelibrary.wiley.com/doi/10.1002/14651858.CD007057.pub3/abstract) (R) | X |  |
|  | [Frenotomy for tongue-tie in newborn infants](http://onlinelibrary.wiley.com/doi/10.1002/14651858.CD011065/abstract) (P) | X |  |
|  | [Chinese herbal medicines for treating osteoporosis](http://onlinelibrary.wiley.com/doi/10.1002/14651858.CD005467.pub2/abstract) (R) | X |  |
|  | [Supportive therapy for schizophrenia](http://onlinelibrary.wiley.com/doi/10.1002/14651858.CD004716.pub3/abstract) (R) | X |  |
|  | [Laparoscopic versus open nephrectomy for live kidney donors](http://onlinelibrary.wiley.com/doi/10.1002/14651858.CD006124.pub2/abstract) (R) | X |  |
|  | [Exercise for osteoarthritis of the hip](http://onlinelibrary.wiley.com/doi/10.1002/14651858.CD007912.pub2/abstract) (R) |  |  |
|  | [Structured treatment interruptions (STI) in chronic suppressed HIV infection in adults](http://onlinelibrary.wiley.com/doi/10.1002/14651858.CD005482/abstract) (R) | X |  |
|  | [Neuromodulators for pain management in rheumatoid arthritis](http://onlinelibrary.wiley.com/doi/10.1002/14651858.CD008921.pub2/abstract) (R) | X |  |
|  | [Biopsy versus resection for high grade glioma](http://onlinelibrary.wiley.com/doi/10.1002/14651858.CD002034/abstract) (R) | X |  |
|  | [Topical umbilical cord care at birth](http://onlinelibrary.wiley.com/doi/10.1002/14651858.CD001057.pub2/abstract) (R) | X |  |
|  | [Seclusion and restraint for people with serious mental illnesses](http://onlinelibrary.wiley.com/doi/10.1002/14651858.CD001163/abstract) (R) | X |  |
|  | [Antifibrinolytics (lysine analogues) for the prevention of bleeding in patients with haematological disorders](http://onlinelibrary.wiley.com/doi/10.1002/14651858.CD009733.pub2/abstract) (R) | X |  |
|  | [The psychological effects of the physical healthcare environment on healthcare personnel](http://onlinelibrary.wiley.com/doi/10.1002/14651858.CD006210.pub3/abstract) (R) | X |  |
|  | [Discharge planning from hospital to home](http://onlinelibrary.wiley.com/doi/10.1002/14651858.CD000313.pub4/abstract) (R) | X |  |
|  | [Fluid replacement therapy for acute episodes of pain in people with sickle cell disease](http://onlinelibrary.wiley.com/doi/10.1002/14651858.CD005406.pub3/abstract) (R) | X |  |
|  | [Interventions for disorders of eye movement in patients with stroke](http://onlinelibrary.wiley.com/doi/10.1002/14651858.CD008389.pub2/abstract) (R) | X |  |
|  | [Cellulose, modified cellulose and synthetic membranes in the haemodialysis of patients with end-stage renal disease](http://onlinelibrary.wiley.com/doi/10.1002/14651858.CD003234.pub2/abstract) (R) | X |  |
|  | [Interventions for smoking cessation in Indigenous populations](http://onlinelibrary.wiley.com/doi/10.1002/14651858.CD009046.pub2/abstract) (R) | X |  |
|  | [Intravenous immunoglobulin for myasthenia gravis](http://onlinelibrary.wiley.com/doi/10.1002/14651858.CD002277.pub4/abstract) (R) | X |  |
|  | [Exercise for osteoarthritis of the knee](http://onlinelibrary.wiley.com/doi/10.1002/14651858.CD004376.pub3/abstract) (R) | X |  |
|  | [Intermittent versus continuous renal replacement therapy for acute renal failure in adults](http://onlinelibrary.wiley.com/doi/10.1002/14651858.CD003773.pub3/abstract) (R) | X |  |
|  | [Physiotherapy for Parkinson's disease: a comparison of techniques](http://onlinelibrary.wiley.com/doi/10.1002/14651858.CD002815.pub2/abstract) (R) | X |  |
|  | [Nonsteroidal anti-inflammatory drugs (NSAIDS) versus opioids for acute renal colic](http://onlinelibrary.wiley.com/doi/10.1002/14651858.CD004137.pub3/abstract) (R) | X |  |
|  | [Risk scoring systems for predicting preterm birth with the aim of reducing associated adverse outcomes](http://onlinelibrary.wiley.com/doi/10.1002/14651858.CD004902.pub4/abstract) (R) | X |  |
|  | [Methods of delivering the placenta at caesarean section](http://onlinelibrary.wiley.com/doi/10.1002/14651858.CD004737.pub2/abstract) (R) | X |  |
|  | [Nortriptyline for neuropathic pain in adults](http://onlinelibrary.wiley.com/doi/10.1002/14651858.CD011209.pub2/abstract) (R) | X |  |
|  | [Topical lidocaine for neuropathic pain in adults](http://onlinelibrary.wiley.com/doi/10.1002/14651858.CD010958.pub2/abstract) (R) | X |  |
|  | [Androgens for the anaemia of chronic kidney disease in adults](http://onlinelibrary.wiley.com/doi/10.1002/14651858.CD006881.pub2/abstract) (R) | X |  |
|  | [Fluticasone versus 'extrafine' HFA-beclomethasone dipropionate for chronic asthma in adults and children](http://onlinelibrary.wiley.com/doi/10.1002/14651858.CD005309.pub3/abstract) (R) | X |  |
|  | [Patient reminder and recall systems to improve immunization rates](http://onlinelibrary.wiley.com/doi/10.1002/14651858.CD003941.pub2/abstract) (R) | X |  |
|  | [Umbilical cord antiseptics for preventing sepsis and death among newborns](http://onlinelibrary.wiley.com/doi/10.1002/14651858.CD008635.pub2/abstract) (R) | X |  |
|  | [Cervico-thoracic or lumbar sympathectomy for neuropathic pain and complex regional pain syndrome](http://onlinelibrary.wiley.com/doi/10.1002/14651858.CD002918.pub3/abstract) (R) | X |  |
|  | [Carotid endarterectomy for symptomatic carotid stenosis](http://onlinelibrary.wiley.com/doi/10.1002/14651858.CD001081.pub2/abstract) (R) | X |  |
|  | [Aripiprazole alone or in combination for acute mania](http://onlinelibrary.wiley.com/doi/10.1002/14651858.CD005000.pub2/abstract) (R) | X |  |
|  | [Minocycline for acne vulgaris: efficacy and safety](http://onlinelibrary.wiley.com/doi/10.1002/14651858.CD002086.pub2/abstract) (R) | X |  |
|  | [Surgical versus non-surgical management of abdominal injury](http://onlinelibrary.wiley.com/doi/10.1002/14651858.CD007383.pub2/abstract) (R) | X |  |
|  | [Hypnosis during pregnancy, childbirth, and the postnatal period for preventing postnatal depression](http://onlinelibrary.wiley.com/doi/10.1002/14651858.CD009062.pub2/abstract) (R) | X |  |
|  | [Interventions for visual field defects in patients with stroke](http://onlinelibrary.wiley.com/doi/10.1002/14651858.CD008388.pub2/abstract) (R) | X |  |
|  | [Exercise for acutely hospitalised older medical patients](http://onlinelibrary.wiley.com/doi/10.1002/14651858.CD005955.pub2/abstract) (R) | X |  |
|  | [Critical incident audit and feedback to improve perinatal and maternal mortality and morbidity](http://onlinelibrary.wiley.com/doi/10.1002/14651858.CD002961.pub2/abstract) (R) | X |  |
|  | [Carnitine supplementation of parenterally fed neonates](http://onlinelibrary.wiley.com/doi/10.1002/14651858.CD000950/abstract) (R) | X |  |
| Fire, Heat, and Hot Substances | [**Steam** inhalation or humidified oxygen for acute bronchiolitis in children up to three years of age](http://onlinelibrary.wiley.com/doi/10.1002/14651858.CD006435.pub2/abstract) (R) | X |  |
|  | [Alcohol **ignition** interlock programmes for reducing drink driving recidivism](http://onlinelibrary.wiley.com/doi/10.1002/14651858.CD004168.pub2/abstract) (R) | X |  |
|  | [Heated, humidified air for the common cold](http://onlinelibrary.wiley.com/doi/10.1002/14651858.CD001728.pub5/abstract) (R) | X |  |
|  | [Heated humidification versus heat and moisture exchangers for ventilated adults and children](http://onlinelibrary.wiley.com/doi/10.1002/14651858.CD004711.pub2/abstract) (R) | X |  |
|  | [Mouthrinses for the treatment of halitosis](http://onlinelibrary.wiley.com/doi/10.1002/14651858.CD006701.pub2/abstract) (R) | X |  |
|  | [Pelvic floor muscle training added to another active treatment versus the same active treatment alone for urinary incontinence in women](http://onlinelibrary.wiley.com/doi/10.1002/14651858.CD010551.pub2/abstract) (R) | X |  |
|  | [Superficial heat or cold for low back pain](http://onlinelibrary.wiley.com/doi/10.1002/14651858.CD004750.pub2/abstract) (R) | X |  |
|  | [Aromatherapy for pain management in labour](http://onlinelibrary.wiley.com/doi/10.1002/14651858.CD009215/abstract) (R) | X |  |
|  | [Acupuncture for Attention Deficit Hyperactivity Disorder (ADHD) in children and adolescents](http://onlinelibrary.wiley.com/doi/10.1002/14651858.CD007839.pub2/abstract) (R) | X |  |
|  | [Pool fencing for preventing drowning of children](http://onlinelibrary.wiley.com/doi/10.1002/14651858.CD001047/abstract) (R) | X |  |
|  | [Acupuncture for uterine fibroids](http://onlinelibrary.wiley.com/doi/10.1002/14651858.CD007221.pub2/abstract) (R) | X |  |
|  | [Interventions for preventing injuries in problem drinkers](http://onlinelibrary.wiley.com/doi/10.1002/14651858.CD001857.pub2/abstract) (R) | X |  |
|  | [Acupuncture for mumps in children](http://onlinelibrary.wiley.com/doi/10.1002/14651858.CD008400.pub3/abstract) (R) | X |  |
|  | [Symptomatic oxygen for non-hypoxaemic chronic obstructive pulmonary disease](http://onlinelibrary.wiley.com/doi/10.1002/14651858.CD006429.pub2/abstract) (R) | X |  |
|  | [Interventions for the treatment of **burning** mouth syndrome](http://onlinelibrary.wiley.com/doi/10.1002/14651858.CD002779.pub2/abstract) (R) | X |  |
|  | [Honey as a topical treatment for wounds](http://onlinelibrary.wiley.com/doi/10.1002/14651858.CD005083.pub3/abstract) (R) | X |  |
|  | [Human albumin solution for resuscitation and volume expansion in critically ill patients](http://onlinelibrary.wiley.com/doi/10.1002/14651858.CD001208.pub4/abstract) (R) | X |  |
|  | [Aloe vera for treating acute and chronic wounds](http://onlinelibrary.wiley.com/doi/10.1002/14651858.CD008762.pub2/abstract) (R) | X |  |
|  | [Hyperbaric oxygen therapy for treating acute surgical and traumatic wounds](http://onlinelibrary.wiley.com/doi/10.1002/14651858.CD008059.pub3/abstract) (R) | X |  |
|  | [Neuromodulators for pain management in rheumatoid arthritis](http://onlinelibrary.wiley.com/doi/10.1002/14651858.CD008921.pub2/abstract) (R) | X |  |
|  | [Hypertonic versus near isotonic crystalloid for fluid resuscitation in critically ill patients](http://onlinelibrary.wiley.com/doi/10.1002/14651858.CD002045.pub2/abstract) (R) | X |  |
|  | [Topical silver for preventing wound infection](http://onlinelibrary.wiley.com/doi/10.1002/14651858.CD006478.pub2/abstract) (R) | X |  |
|  | [Topical capsaicin (high concentration) for chronic neuropathic pain in adults](http://onlinelibrary.wiley.com/doi/10.1002/14651858.CD007393.pub3/abstract) (R) | X |  |
|  | [Aromatherapy for dementia](http://onlinelibrary.wiley.com/doi/10.1002/14651858.CD003150.pub2/abstract) (R) | X |  |
|  | [Nutritional support for critically ill children](http://onlinelibrary.wiley.com/doi/10.1002/14651858.CD005144.pub2/abstract) (R) | X |  |
|  | [Topical treatments for chronic plaque psoriasis](http://onlinelibrary.wiley.com/doi/10.1002/14651858.CD005028.pub3/abstract) (R) | X |  |
|  | [Silicone gel sheeting for preventing and treating hypertrophic and keloid scars](http://onlinelibrary.wiley.com/doi/10.1002/14651858.CD003826.pub3/abstract) (R) | X |  |
|  | [Topical interventions for genital lichen sclerosus](http://onlinelibrary.wiley.com/doi/10.1002/14651858.CD008240.pub2/abstract) (R) | X |  |
|  | [Oral rinses, mouthwashes and sprays for improving recovery following tonsillectomy](http://onlinelibrary.wiley.com/doi/10.1002/14651858.CD007806.pub4/abstract) (R) | X |  |
|  | [Double wall versus single wall incubator for reducing heat loss in very low birth weight infants in incubators](http://onlinelibrary.wiley.com/doi/10.1002/14651858.CD004215.pub2/abstract) (R) | X |  |
|  | [Surgical interventions for high-grade vulval intraepithelial neoplasia](http://onlinelibrary.wiley.com/doi/10.1002/14651858.CD007928.pub3/abstract) (R) | X |  |
|  | [Laser and photoepilation for unwanted hair growth](http://onlinelibrary.wiley.com/doi/10.1002/14651858.CD004684.pub2/abstract) (R) | X |  |
|  | [Topical pimecrolimus for eczema](http://onlinelibrary.wiley.com/doi/10.1002/14651858.CD005500.pub2/abstract) (R) | X |  |
|  | [Colloids versus crystalloids for fluid resuscitation in critically ill patients](http://onlinelibrary.wiley.com/doi/10.1002/14651858.CD000567.pub6/abstract) (R) | X |  |
|  | [Antibiotic therapy for the treatment of methicillin-resistant Staphylococcus aureus (MRSA) in non surgical wounds](http://onlinelibrary.wiley.com/doi/10.1002/14651858.CD010427.pub2/abstract) (R) | X |  |
|  | [Vitamin C for preventing and treating pneumonia](http://onlinelibrary.wiley.com/doi/10.1002/14651858.CD005532.pub3/abstract) (R) | X |  |
|  | [Oestrogens for preventing recurrent urinary tract infection in postmenopausal women](http://onlinelibrary.wiley.com/doi/10.1002/14651858.CD005131.pub2/abstract) (R) | X |  |
|  | [Cephalic version by moxibustion for breech presentation](http://onlinelibrary.wiley.com/doi/10.1002/14651858.CD003928.pub3/abstract) (R) | X |  |
|  | [Interventions for toxic epidermal necrolysis](http://onlinelibrary.wiley.com/doi/10.1002/14651858.CD001435/abstract) (R) | X |  |
|  | [Routine perineal shaving on admission in labour](http://onlinelibrary.wiley.com/doi/10.1002/14651858.CD001236.pub2/abstract) (R) | X |  |
|  | [Tramadol for neuropathic pain](http://onlinelibrary.wiley.com/doi/10.1002/14651858.CD003726.pub3/abstract) (R) | X |  |
|  | [Duration of antibacterial treatment for uncomplicated urinary tract infection in women](http://onlinelibrary.wiley.com/doi/10.1002/14651858.CD004682.pub2/abstract) (R) | X |  |
|  | [5-FU for genital warts in non-immunocompromised individuals](http://onlinelibrary.wiley.com/doi/10.1002/14651858.CD006562.pub2/abstract) (R) | X |  |
|  | [Monosodium glutamate avoidance for chronic asthma in adults and children](http://onlinelibrary.wiley.com/doi/10.1002/14651858.CD004357.pub4/abstract) (R) | X |  |
|  | [Interventions for impetigo](http://onlinelibrary.wiley.com/doi/10.1002/14651858.CD003261.pub3/abstract) (R) | X |  |
|  | [Physical training for McArdle disease](http://onlinelibrary.wiley.com/doi/10.1002/14651858.CD007931.pub2/abstract) (R) | X |  |
|  | [N-acetylcysteine for sepsis and systemic inflammatory response in adults](http://onlinelibrary.wiley.com/doi/10.1002/14651858.CD006616.pub2/abstract) (R) | X |  |
|  | [Interventions for the management of oral submucous fibrosis](http://onlinelibrary.wiley.com/doi/10.1002/14651858.CD007156.pub2/abstract) (R) | X |  |
|  | [Topical capsaicin (low concentration) for chronic neuropathic pain in adults](http://onlinelibrary.wiley.com/doi/10.1002/14651858.CD010111/abstract) (R) | X |  |
|  | [Punctal occlusion for dry eye syndrome](http://onlinelibrary.wiley.com/doi/10.1002/14651858.CD006775.pub2/abstract) (R) | X |  |
|  | [Minimally invasive discectomy versus microdiscectomy/open discectomy for symptomatic lumbar disc herniation](http://onlinelibrary.wiley.com/doi/10.1002/14651858.CD010328.pub2/abstract) (R) | X |  |
|  | [Interventions for chronic blepharitis](http://onlinelibrary.wiley.com/doi/10.1002/14651858.CD005556.pub2/abstract) (R) | X |  |
|  | [Topical anti-inflammatory agents for seborrhoeic dermatitis of the face or scalp](http://onlinelibrary.wiley.com/doi/10.1002/14651858.CD009446.pub2/abstract) (R) | X |  |
|  | [Local cooling for relieving pain from perineal trauma sustained during childbirth](http://onlinelibrary.wiley.com/doi/10.1002/14651858.CD006304.pub3/abstract) (R) | X |  |
|  | [Sclerotherapy for lower limb telangiectasias](http://onlinelibrary.wiley.com/doi/10.1002/14651858.CD008826.pub2/abstract) (R) | X |  |
|  | [Laparoscopic surgery for endometriosis](http://onlinelibrary.wiley.com/doi/10.1002/14651858.CD011031.pub2/abstract) (R) | X |  |
|  | [Sumatriptan plus naproxen for acute migraine attacks in adults](http://onlinelibrary.wiley.com/doi/10.1002/14651858.CD008541.pub2/abstract) (R) | X |  |
|  | [Injection sclerotherapy for varicose veins](http://onlinelibrary.wiley.com/doi/10.1002/14651858.CD001732.pub2/abstract) (R) | X |  |
|  | [Interventions for melasma](http://onlinelibrary.wiley.com/doi/10.1002/14651858.CD003583.pub2/abstract) (R) | X |  |
|  | [Conservative management of symptomatic and/or complicated haemorrhoids in pregnancy and the puerperium](http://onlinelibrary.wiley.com/doi/10.1002/14651858.CD004077.pub2/abstract) (R) | X |  |
|  | [Techniques for the interruption of tubal patency for female sterilisation](http://onlinelibrary.wiley.com/doi/10.1002/14651858.CD003034.pub2/abstract) (R) | X |  |
|  | [Treatment for Barrett's oesophagus](http://onlinelibrary.wiley.com/doi/10.1002/14651858.CD004060.pub2/abstract) (R) | X |  |
|  | [Topical herbal therapies for treating osteoarthritis](http://onlinelibrary.wiley.com/doi/10.1002/14651858.CD010538/abstract) (R) | X |  |
|  | [Prophylaxis for venous thromboembolic disease in pregnancy and the early postnatal period](http://onlinelibrary.wiley.com/doi/10.1002/14651858.CD001689.pub3/abstract) (R) | X |  |
|  | [Interventions for photodamaged skin](http://onlinelibrary.wiley.com/doi/10.1002/14651858.CD001782.pub2/abstract) (R) | X |  |
|  | [Bisphosphonate therapy for children and adolescents with secondary osteoporosis](http://onlinelibrary.wiley.com/doi/10.1002/14651858.CD005324.pub2/abstract) (R) | X |  |
|  | [Interventions for erosive lichen planus affecting mucosal sites](http://onlinelibrary.wiley.com/doi/10.1002/14651858.CD008092.pub2/abstract) (R) | X |  |
|  | [Interventions for mycosis fungoides](http://onlinelibrary.wiley.com/doi/10.1002/14651858.CD008946.pub2/abstract) (R) | X |  |
|  | [Selenium supplementation for critically ill adults](http://onlinelibrary.wiley.com/doi/10.1002/14651858.CD003703.pub2/abstract) (R) | X |  |
|  | [Medical interventions for high grade vulval intraepithelial neoplasia](http://onlinelibrary.wiley.com/doi/10.1002/14651858.CD007924.pub2/abstract) (R) | X |  |
|  | [Rutosides for treatment of post-thrombotic syndrome](http://onlinelibrary.wiley.com/doi/10.1002/14651858.CD005625.pub2/abstract) (R) | X |  |
|  | [Stretch for the treatment and prevention of contractures](http://onlinelibrary.wiley.com/doi/10.1002/14651858.CD007455.pub2/abstract) (R) | X |  |
|  | [Topical agents or dressings for pain in venous leg ulcers](http://onlinelibrary.wiley.com/doi/10.1002/14651858.CD001177.pub3/abstract) (R) | X |  |
|  | [Interventions for promoting physical activity in people with cystic fibrosis](http://onlinelibrary.wiley.com/doi/10.1002/14651858.CD009448.pub2/abstract) (R) | X |  |
|  | [Acupuncture for neck disorders](http://onlinelibrary.wiley.com/doi/10.1002/14651858.CD004870.pub3/abstract) (R) | X |  |
|  | [Diazepam for treating tetanus](http://onlinelibrary.wiley.com/doi/10.1002/14651858.CD003954.pub2/abstract) (R) | X |  |
|  | [Topical antifungal treatments for tinea cruris and tinea corporis](http://onlinelibrary.wiley.com/doi/10.1002/14651858.CD009992.pub2/abstract) (R) | X |  |
|  | [Antimicrobial agents for treating uncomplicated urinary tract infection in women](http://onlinelibrary.wiley.com/doi/10.1002/14651858.CD007182.pub2/abstract) (R) | X |  |
|  | [Crisis intervention for people with severe mental illnesses](http://onlinelibrary.wiley.com/doi/10.1002/14651858.CD001087.pub4/abstract) (R) | X |  |
|  | [Optical coherence tomography (OCT) for detection of macular oedema in patients with diabetic retinopathy](http://onlinelibrary.wiley.com/doi/10.1002/14651858.CD008081.pub3/abstract) (R) | X |  |
| Poisoning | [Laetrile treatment for cancer](http://onlinelibrary.wiley.com/doi/10.1002/14651858.CD005476.pub3/abstract) (R) | X |  |
|  | [Hyperbaric oxygen therapy for the adjunctive treatment of traumatic brain injury](http://onlinelibrary.wiley.com/doi/10.1002/14651858.CD004609.pub3/abstract) (R) | X |  |
|  | [Prophylactic antibiotics for preventing pneumococcal infection in children with sickle cell disease](http://onlinelibrary.wiley.com/doi/10.1002/14651858.CD003427.pub3/abstract) (R) | X |  |
|  | [Psychosocial and pharmacological treatments for deliberate self harm](http://onlinelibrary.wiley.com/doi/10.1002/14651858.CD001764/abstract) (R) | X |  |
|  | [D-Penicillamine for preventing retinopathy of prematurity in preterm infants](http://onlinelibrary.wiley.com/doi/10.1002/14651858.CD001073.pub2/abstract) (R) | X |  |
|  | [Vaccines for preventing plague](http://onlinelibrary.wiley.com/doi/10.1002/14651858.CD000976/abstract) (R) | X |  |
| Mechanical Forces | Hydroxyethyl starch (HES) versus other fluid therapies: effects on kidney function (R) | X |  |
|  | Interventions for protecting renal function in the perioperative period (R) | X |  |
|  | [Interventions for preventing blood loss during the treatment of cervical intraepithelial neoplasia](http://onlinelibrary.wiley.com/doi/10.1002/14651858.CD001421.pub3/abstract) (R) | X |  |
|  | [Surgery for cervical intraepithelial neoplasia](http://onlinelibrary.wiley.com/doi/10.1002/14651858.CD001318.pub3/abstract) (R) | X |  |
|  | [Interventions for treating brain arteriovenous malformations in adults](http://onlinelibrary.wiley.com/doi/10.1002/14651858.CD003436.pub3/abstract) (R) | X |  |
|  | [Anticonvulsants for fibromyalgia](http://onlinelibrary.wiley.com/doi/10.1002/14651858.CD010782/abstract) (R) | X |  |
|  | [Surgery versus radiosurgery for patients with a solitary brain metastasis from non-small cell lung cancer](http://onlinelibrary.wiley.com/doi/10.1002/14651858.CD004840.pub2/abstract) (R) | X |  |
|  | [Selective computed tomography (CT) versus routine thoracoabdominal CT for high-energy blunt-trauma patients](http://onlinelibrary.wiley.com/doi/10.1002/14651858.CD009743.pub2/abstract) (R) | X |  |
|  | [Neurosurgical interventions for the treatment of classical trigeminal neuralgia](http://onlinelibrary.wiley.com/doi/10.1002/14651858.CD007312.pub2/abstract) (R) | X |  |
|  | [Inferior turbinate surgery for nasal obstruction in allergic rhinitis after failed medical treatment](http://onlinelibrary.wiley.com/doi/10.1002/14651858.CD005235.pub2/abstract) (R) | X |  |
|  | [Non-antiepileptic drugs for trigeminal neuralgia](http://onlinelibrary.wiley.com/doi/10.1002/14651858.CD004029.pub4/abstract) (R) | X |  |
|  | [Ultrasound use for the placement of haemodialysis catheters](http://onlinelibrary.wiley.com/doi/10.1002/14651858.CD005279.pub4/abstract) (R) | X |  |
|  | [Surgical interventions for treating acute Achilles tendon ruptures](http://onlinelibrary.wiley.com/doi/10.1002/14651858.CD003674.pub4/abstract) (R) | X |  |
|  | [Surgical versus non-surgical management of abdominal injury](http://onlinelibrary.wiley.com/doi/10.1002/14651858.CD007383.pub2/abstract) (R) | X |  |
|  | [Unfractionated heparin versus low molecular weight heparin for avoiding heparin-induced thrombocytopenia in postoperative patients](http://onlinelibrary.wiley.com/doi/10.1002/14651858.CD007557.pub2/abstract) (R) | X |  |
|  | [Systemic treatments for the prevention of venous thrombo-embolic events in paediatric cancer patients with tunnelled central venous catheters](http://onlinelibrary.wiley.com/doi/10.1002/14651858.CD009160.pub2/abstract) (R) | X |  |
|  | [Publication bias in clinical trials due to statistical significance or direction of trial results](http://onlinelibrary.wiley.com/doi/10.1002/14651858.MR000006.pub3/abstract) (R) | X |  |
|  | [Autologous hematopoietic stem cell transplantation following high dose chemotherapy for non-rhabdomyosarcoma soft tissue sarcomas](http://onlinelibrary.wiley.com/doi/10.1002/14651858.CD008216.pub4/abstract) (R) | X |  |
|  | [Heparin versus normal saline for patency of arterial lines](http://onlinelibrary.wiley.com/doi/10.1002/14651858.CD007364.pub2/abstract) (R) | X |  |
|  | [Paying for performance to improve the delivery of health interventions in low- and middle-income countries](http://onlinelibrary.wiley.com/doi/10.1002/14651858.CD007899.pub2/abstract) (R) | X |  |
|  | [Face-to-face versus remote and web 2.0 interventions for promoting physical activity](http://onlinelibrary.wiley.com/doi/10.1002/14651858.CD010393.pub2/abstract) (R) | X |  |
|  | [Abdominal decompression for suspected fetal compromise/pre-eclampsia](http://onlinelibrary.wiley.com/doi/10.1002/14651858.CD000004.pub2/abstract) (R) | X |  |
|  | [Insoles for prevention and treatment of back pain](http://onlinelibrary.wiley.com/doi/10.1002/14651858.CD005275.pub2/abstract) (R) | X |  |
|  | [Distal aortic perfusion during thoracoabdominal aneurysm repair for prevention of paraplegia](http://onlinelibrary.wiley.com/doi/10.1002/14651858.CD008197.pub2/abstract) (R) | X |  |
|  | [Antimicrobial agents for preventing peritonitis in peritoneal dialysis patients](http://onlinelibrary.wiley.com/doi/10.1002/14651858.CD004679.pub2/abstract) (R) | X |  |
|  | [Percussion, diuresis, and inversion therapy for the passage of lower pole kidney stones following shock wave lithotripsy](http://onlinelibrary.wiley.com/doi/10.1002/14651858.CD008569.pub2/abstract) (R) | X |  |
|  | [Time to publication for results of clinical trials](http://onlinelibrary.wiley.com/doi/10.1002/14651858.MR000011.pub2/abstract) (R) | X |  |
|  | [Surgery for faecal incontinence in adults](http://onlinelibrary.wiley.com/doi/10.1002/14651858.CD001757.pub4/abstract) (R) | X |  |
|  | [Orthodontic and orthopaedic treatment for anterior open **bite** in children](http://onlinelibrary.wiley.com/doi/10.1002/14651858.CD005515.pub3/abstract) (R) | X |  |
|  | [Electronic mosquito repellents for preventing mosquito **bites** and malaria infection](http://onlinelibrary.wiley.com/doi/10.1002/14651858.CD005434.pub2/abstract) (R) | X |  |
|  | [Orthodontic treatment for deep **bite** and retroclined upper front teeth in children](http://onlinelibrary.wiley.com/doi/10.1002/14651858.CD005972.pub2/abstract) (R) | X |  |
|  | [Primary closure versus delayed closure for non **bite** traumatic wounds within 24 hours post injury](http://onlinelibrary.wiley.com/doi/10.1002/14651858.CD008574.pub3/abstract) (R) | X |  |
|  | [Occlusal adjustment for treating and preventing temporomandibular joint disorders](http://onlinelibrary.wiley.com/doi/10.1002/14651858.CD003812/abstract) (R) | X |  |
|  | [Orthodontic treatment for posterior crossbites](http://onlinelibrary.wiley.com/doi/10.1002/14651858.CD000979.pub2/abstract) (R) | X |  |
|  | [Sealants for preventing dental decay in the permanent teeth](http://onlinelibrary.wiley.com/doi/10.1002/14651858.CD001830.pub4/abstract) (R) | X |  |
|  | [Intraoperative local anaesthesia for reduction of postoperative pain following general anaesthesia for dental treatment in children and adolescents](http://onlinelibrary.wiley.com/doi/10.1002/14651858.CD009742.pub2/abstract) (R) | X |  |
|  | [Stabilisation splint therapy for temporomandibular pain dysfunction syndrome](http://onlinelibrary.wiley.com/doi/10.1002/14651858.CD002778.pub2/abstract) (R) | X |  |
|  | [Primaquine or other 8-aminoquinoline for reducing *Plasmodium falciparum* transmission](http://onlinelibrary.wiley.com/doi/10.1002/14651858.CD008152.pub4/abstract) (R) | X |  |
|  | [Orthodontic treatment for prominent lower front teeth (Class III malocclusion) in children](http://onlinelibrary.wiley.com/doi/10.1002/14651858.CD003451.pub2/abstract) (R) | X |  |
|  | [Oral traditional Chinese medication for adhesive small bowel obstruction](http://onlinelibrary.wiley.com/doi/10.1002/14651858.CD008836.pub2/abstract) (R) | X |  |
|  | [Drug treatment for spinal muscular atrophy types II and III](http://onlinelibrary.wiley.com/doi/10.1002/14651858.CD006282.pub4/abstract) (R) | X |  |
|  | [Interventions for preventing reactions to snake antivenom](http://onlinelibrary.wiley.com/doi/10.1002/14651858.CD002153/abstract) (R) | X |  |
|  | [Venom immunotherapy for preventing allergic reactions to insect stings](http://onlinelibrary.wiley.com/doi/10.1002/14651858.CD008838.pub2/abstract) (R) | X |  |
|  | [Interventions for the symptoms and signs resulting from jellyfish stings](http://onlinelibrary.wiley.com/doi/10.1002/14651858.CD009688.pub2/abstract) (R) | X |  |
|  | [Orthodontics for treating temporomandibular joint (TMJ) disorders](http://onlinelibrary.wiley.com/doi/10.1002/14651858.CD006541.pub2/abstract) (R) | X |  |
|  | [Preoperative analgesics for additional pain relief in children and adolescents having dental treatment](http://onlinelibrary.wiley.com/doi/10.1002/14651858.CD008392.pub2/abstract) (R) | X |  |
|  | [Artemisinin-based combination therapy for treating uncomplicated malaria](http://onlinelibrary.wiley.com/doi/10.1002/14651858.CD007483.pub2/abstract) (R) | X |  |
|  | [Silicone gel sheeting for preventing and treating hypertrophic and keloid scars](http://onlinelibrary.wiley.com/doi/10.1002/14651858.CD003826.pub3/abstract) (R) | X |  |
|  | [Systemic antibiotics for symptomatic apical periodontitis and acute apical abscess in adults](http://onlinelibrary.wiley.com/doi/10.1002/14651858.CD010136.pub2/abstract) (R) | X |  |
|  | [Ivermectin for onchocercal eye disease (river blindness)](http://onlinelibrary.wiley.com/doi/10.1002/14651858.CD002219.pub2/abstract) (R) | X |  |
|  | [Iron-chelating agents for treating malaria](http://onlinelibrary.wiley.com/doi/10.1002/14651858.CD001474/abstract) (R) | X |  |
|  | [Botulinum toxin for masseter hypertrophy](http://onlinelibrary.wiley.com/doi/10.1002/14651858.CD007510.pub3/abstract) (R) | X |  |
|  | [Antibiotics for acute bronchitis](http://onlinelibrary.wiley.com/doi/10.1002/14651858.CD000245.pub3/abstract) (R) | X |  |
|  | [Interventions for treating traumatised permanent front teeth: luxated (dislodged) teeth](http://onlinelibrary.wiley.com/doi/10.1002/14651858.CD006203.pub2/abstract) (R) | X |  |
|  | [Interventions for the treatment of fractures of the mandibular condyle](http://onlinelibrary.wiley.com/doi/10.1002/14651858.CD006538.pub2/abstract) (R) | X |  |
|  | [Artemether for severe malaria](http://onlinelibrary.wiley.com/doi/10.1002/14651858.CD010678.pub2/abstract) (R) | X |  |
|  | [Chinese herbal medicines for treating skin and soft-tissue infections](http://onlinelibrary.wiley.com/doi/10.1002/14651858.CD010619.pub2/abstract) (R) | X |  |
|  | [Antibiotics for acute maxillary sinusitis in adults](http://onlinelibrary.wiley.com/doi/10.1002/14651858.CD000243.pub3/abstract) (R) | X |  |
|  | [Vaccines for preventing tick-borne encephalitis](http://onlinelibrary.wiley.com/doi/10.1002/14651858.CD000977.pub2/abstract) (R) | X |  |
|  | [Chemotherapy for second-stage Human African trypanosomiasis](http://onlinelibrary.wiley.com/doi/10.1002/14651858.CD006201.pub3/abstract) (R) | X |  |
|  | [Honey as a topical treatment for wounds](http://onlinelibrary.wiley.com/doi/10.1002/14651858.CD005083.pub3/abstract) (R) | X |  |
|  | [Laryngeal mask airway versus endotracheal tube for percutaneous dilatational tracheostomy in critically ill adult patients](http://onlinelibrary.wiley.com/doi/10.1002/14651858.CD009901.pub2/abstract) (R) | X |  |
|  | [Botulinum toxin for upper oesophageal sphincter dysfunction in neurological swallowing disorders](http://onlinelibrary.wiley.com/doi/10.1002/14651858.CD009968.pub2/abstract) (R) | X |  |
|  | [Non-pharmacological therapies for dysphagia in Parkinson's disease](http://onlinelibrary.wiley.com/doi/10.1002/14651858.CD002816/abstract) (R) | X |  |
|  | [Pain relief for neonatal circumcision](http://onlinelibrary.wiley.com/doi/10.1002/14651858.CD004217.pub2/abstract) (R) | X |  |
|  | [Techniques for the interruption of tubal patency for female sterilisation](http://onlinelibrary.wiley.com/doi/10.1002/14651858.CD003034.pub2/abstract) (R) | X |  |
|  | [Honey and lozenges for children with non-specific cough](http://onlinelibrary.wiley.com/doi/10.1002/14651858.CD007523.pub2/abstract) (R) | X |  |
|  | [Immunosuppressive agents for myasthenia gravis](http://onlinelibrary.wiley.com/doi/10.1002/14651858.CD005224.pub2/abstract) (R) | X |  |
|  | [Alternative injectable materials for vocal fold medialisation in unilateral vocal fold paralysis](http://onlinelibrary.wiley.com/doi/10.1002/14651858.CD009239.pub2/abstract) (R) | X |  |
|  | [Electrotherapy for neck pain](http://onlinelibrary.wiley.com/doi/10.1002/14651858.CD004251.pub5/abstract) (R) | X |  |
|  | [Electrical stimulation with non-implanted electrodes for urinary incontinence in men](http://onlinelibrary.wiley.com/doi/10.1002/14651858.CD001202.pub5/abstract) (R) | X |  |
|  | [Acupuncture and related interventions for smoking cessation](http://onlinelibrary.wiley.com/doi/10.1002/14651858.CD000009.pub4/abstract) (R) | X |  |
|  | [Radiofrequency (thermal) ablation versus no intervention or other interventions for hepatocellular carcinoma](http://onlinelibrary.wiley.com/doi/10.1002/14651858.CD003046.pub3/abstract) (R) | X |  |
|  | [Acupuncture and electroacupuncture for the treatment of rheumatoid arthritis](http://onlinelibrary.wiley.com/doi/10.1002/14651858.CD003788.pub2/abstract) (R) | X |  |
|  | [Radiofrequency denervation for neck and back pain](http://onlinelibrary.wiley.com/doi/10.1002/14651858.CD004058/abstract) (R) | X |  |
|  | [Transcutaneous electrical nerve stimulation for primary dysmenorrhoea](http://onlinelibrary.wiley.com/doi/10.1002/14651858.CD002123/abstract) (R) | X |  |
|  | Cardiopulmonary resuscitation (CPR) plus delayed defibrillation versus immediate defibrillation for out-of-hospital cardiac arrest (R) | X |  |
|  | Benzodiazepines for catatonia in people with schizophrenia and other serious mental illnesses (R) | X |  |
|  | Pharmacological cardioversion for atrial fibrillation and flutter (R) | X |  |
|  | Alarm interventions for nocturnal enuresis in children (R) | X |  |
|  | Aerosolized prostacyclin for acute lung injury (ALI) and acute respiratory distress syndrome (ARDS) (R) | X |  |
|  | Social norms information for alcohol misuse in university and college students (R) | X |  |
| Adverse Effects of Medical Treatment | [Industry sponsorship and research outcome](http://onlinelibrary.wiley.com/doi/10.1002/14651858.MR000033.pub2/abstract) (R) | X |  |
|  | [Interventions for preventing and reducing the use of physical restraints in long-term geriatric care](http://onlinelibrary.wiley.com/doi/10.1002/14651858.CD007546.pub2/abstract) (R) | X |  |
|  | [Infection control strategies for preventing the transmission of meticillin-resistant *Staphylococcus aureus* (MRSA) in nursing homes for older people](http://onlinelibrary.wiley.com/doi/10.1002/14651858.CD006354.pub4/abstract) (R) | X |  |
|  | [Arthroplasty versus fusion in single-level cervical degenerative disc disease](http://onlinelibrary.wiley.com/doi/10.1002/14651858.CD009173.pub2/abstract) (R) | X |  |
|  | [Amplification with hearing aids for patients with tinnitus and co-existing hearing loss](http://onlinelibrary.wiley.com/doi/10.1002/14651858.CD010151.pub2/abstract) (R) | X |  |
|  | [Treatment of recurrent stress urinary incontinence after failed minimally invasive synthetic suburethral tape surgery in women](http://onlinelibrary.wiley.com/doi/10.1002/14651858.CD009407.pub2/abstract) (R) | X |  |
|  | [Non-surgical interventions for paediatric pes planus](http://onlinelibrary.wiley.com/doi/10.1002/14651858.CD006311.pub2/abstract) (R) | X |  |
|  | [Sedative techniques for endoscopic retrograde cholangiopancreatography](http://onlinelibrary.wiley.com/doi/10.1002/14651858.CD007274.pub2/abstract) (R) | X |  |
|  | [Enzyme replacement therapy with idursulfase for mucopolysaccharidosis type II (Hunter syndrome)](http://onlinelibrary.wiley.com/doi/10.1002/14651858.CD008185.pub3/abstract) (R) | X |  |
|  | [Recombinant human thyrotropin (rhTSH) aided radioiodine treatment for residual or metastatic differentiated thyroid cancer](http://onlinelibrary.wiley.com/doi/10.1002/14651858.CD008302.pub2/abstract) (R) | X |  |
|  | [Peginterferon plus ribavirin versus interferon plus ribavirin for chronic hepatitis (R)](http://onlinelibrary.wiley.com/doi/10.1002/14651858.CD005441.pub3/abstract) | X |  |
| Animal Contact | [Educational games for health professionals](http://onlinelibrary.wiley.com/doi/10.1002/14651858.CD006411.pub4/abstract) (R) | X |  |
|  | [Chinese herbal medicines for treating osteoporosis](http://onlinelibrary.wiley.com/doi/10.1002/14651858.CD005467.pub2/abstract) (R) | X |  |
|  | [Complementary therapies for acne vulgaris](http://onlinelibrary.wiley.com/doi/10.1002/14651858.CD009436.pub2/abstract) (R) | X |  |
|  | [Sclerotherapy for lower limb telangiectasias](http://onlinelibrary.wiley.com/doi/10.1002/14651858.CD008826.pub2/abstract) (R) | X |  |
|  | [Anticonvulsants for alcohol withdrawal](http://onlinelibrary.wiley.com/doi/10.1002/14651858.CD005064.pub3/abstract) (R) | X |  |
|  | [Virtual reality for treatment compliance for people with serious mental illness](http://onlinelibrary.wiley.com/doi/10.1002/14651858.CD009928.pub2/abstract) (R) | X |  |
|  | Cordyceps sinensis (a traditional Chinese medicine) for treating chronic kidney disease (R) | X |  |
|  | Immediate versus deferred delivery of the preterm baby with suspected fetal compromise for improving outcomes (R) | X |  |
|  | [Vaginal chlorhexidine during labour to prevent early-onset neonatal group B streptococcal infection](http://onlinelibrary.wiley.com/doi/10.1002/14651858.CD003520.pub3/abstract) (R) | X |  |
|  | [Oral rinses, mouthwashes and sprays for improving recovery following tonsillectomy](http://onlinelibrary.wiley.com/doi/10.1002/14651858.CD007806.pub4/abstract) (R) | X |  |
|  | [Topical herbal therapies for treating osteoarthritis](http://onlinelibrary.wiley.com/doi/10.1002/14651858.CD010538/abstract) (R) | X |  |
|  | [Mohs micrographic surgery versus surgical excision for periocular basal cell carcinoma](http://onlinelibrary.wiley.com/doi/10.1002/14651858.CD007041.pub4/abstract) (R) | X |  |
|  | [Topical capsaicin (low concentration) for chronic neuropathic pain in adults](http://onlinelibrary.wiley.com/doi/10.1002/14651858.CD010111/abstract) (R) | X |  |
|  | [Electronic mosquito repellents for preventing mosquito bites and malaria infection](http://onlinelibrary.wiley.com/doi/10.1002/14651858.CD005434.pub2/abstract) (R) | X |  |
|  | [Topical cyclosporine for atopic keratoconjunctivitis](http://onlinelibrary.wiley.com/doi/10.1002/14651858.CD009078.pub2/abstract) (R) | X |  |
|  | [Adrenaline auto-injectors for the treatment of anaphylaxis with and without cardiovascular collapse in the community](http://onlinelibrary.wiley.com/doi/10.1002/14651858.CD008935.pub2/abstract) (R) | X |  |
|  | [Interventions for melasma](http://onlinelibrary.wiley.com/doi/10.1002/14651858.CD003583.pub2/abstract) (R) | X |  |
|  | [Interventions for photodamaged skin](http://onlinelibrary.wiley.com/doi/10.1002/14651858.CD001782.pub2/abstract) (R) | X |  |
|  | [Adrenaline (epinephrine) for the treatment of anaphylaxis with and without shock](http://onlinelibrary.wiley.com/doi/10.1002/14651858.CD006312.pub2/abstract) (R) |  |  |
|  | [Glucocorticoids for the treatment of anaphylaxis](http://onlinelibrary.wiley.com/doi/10.1002/14651858.CD007596.pub3/abstract) (R) | X |  |
|  | [Interventions for preventing occupational irritant hand dermatitis](http://onlinelibrary.wiley.com/doi/10.1002/14651858.CD004414.pub2/abstract) (R) | X |  |
|  | [Percutaneous needle aspiration, injection, and re-aspiration with or without benzimidazole coverage for uncomplicated hepatic hydatid cysts](http://onlinelibrary.wiley.com/doi/10.1002/14651858.CD003623.pub3/abstract) (R) | X |  |
|  | Antiviral treatment for Bell's palsy (idiopathic facial paralysis) (R) | X |  |
|  | Physical therapy for Bell's palsy (idiopathic facial paralysis) (R) | X |  |
|  | Interventions for Bell's Palsy (idiopathic facial paralysis) (P) | X |  |
| Self-harm | [Pharmacological interventions for borderline personality disorder](http://onlinelibrary.wiley.com/doi/10.1002/14651858.CD005653.pub2/abstract) (R) | X |  |
|  | [Advance treatment directives for people with severe mental illness](http://onlinelibrary.wiley.com/doi/10.1002/14651858.CD005963.pub2/abstract) (R) | X |  |
|  | [Length of hospitalisation for people with severe mental illness](http://onlinelibrary.wiley.com/doi/10.1002/14651858.CD000384.pub3/abstract) (R) | X |  |
|  | [Psychological therapies for people with borderline personality disorder](http://onlinelibrary.wiley.com/doi/10.1002/14651858.CD005652.pub2/abstract) (R) | X |  |
|  | [Containment strategies for people with serious mental illness](http://onlinelibrary.wiley.com/doi/10.1002/14651858.CD002084.pub2/abstract) (R) | X |  |
|  | [Crisis interventions for people with borderline personality disorder](http://onlinelibrary.wiley.com/doi/10.1002/14651858.CD009353.pub2/abstract) (R) | X |  |
|  | [Olanzapine IM or velotab for acutely disturbed/agitated people with suspected serious mental illnesses](http://onlinelibrary.wiley.com/doi/10.1002/14651858.CD003729.pub2/abstract) (R) | X |  |
|  | [Lithium for maintenance treatment of mood disorders](http://onlinelibrary.wiley.com/doi/10.1002/14651858.CD003013/abstract) (R) | X |  |
|  | [Psychological and educational interventions for preventing depression in children and adolescents](http://onlinelibrary.wiley.com/doi/10.1002/14651858.CD003380.pub3/abstract) (R) | X |  |
|  | [Tricyclic drugs for depression in children and adolescents](http://onlinelibrary.wiley.com/doi/10.1002/14651858.CD002317.pub2/abstract) (R) | X |  |
|  | [Newer generation antidepressants for depressive disorders in children and adolescents](http://onlinelibrary.wiley.com/doi/10.1002/14651858.CD004851.pub3/abstract) (R) | X |  |
|  | [Lithium for maintenance treatment of mood disorders](http://onlinelibrary.wiley.com/doi/10.1002/14651858.CD003013/abstract) (R) | X |  |
|  | [Community mental health teams (CMHTs) for people with severe mental illnesses and disordered personality](http://onlinelibrary.wiley.com/doi/10.1002/14651858.CD000270.pub2/abstract) (R) | X |  |
|  | [Interventions for preventing injuries in problem drinkers](http://onlinelibrary.wiley.com/doi/10.1002/14651858.CD001857.pub2/abstract) (R) | X |  |
|  | [Psychological therapies versus antidepressant medication, alone and in combination for depression in children and adolescents](http://onlinelibrary.wiley.com/doi/10.1002/14651858.CD008324.pub3/abstract) (R) | X |  |
|  | [Intensive case management for severe mental illness](http://onlinelibrary.wiley.com/doi/10.1002/14651858.CD007906.pub2/abstract) (R) | X |  |
|  | [Pharmacological interventions for those who have sexually offended or are at risk of offending](http://onlinelibrary.wiley.com/doi/10.1002/14651858.CD007989.pub2/abstract) (R) | X |  |
|  | [Motivational interviewing for alcohol misuse in young adults](http://onlinelibrary.wiley.com/doi/10.1002/14651858.CD007025.pub2/abstract) (R) | X |  |
|  | [Quetiapine versus typical antipsychotic medications for schizophrenia](http://onlinelibrary.wiley.com/doi/10.1002/14651858.CD007815.pub2/abstract) (R) | X |  |
|  | [Drugs for preventing malaria in travellers](http://onlinelibrary.wiley.com/doi/10.1002/14651858.CD006491.pub2/abstract) (R) | X |  |
|  | [Psychosocial interventions for people with both severe mental illness and substance misuse](http://onlinelibrary.wiley.com/doi/10.1002/14651858.CD001088.pub3/abstract) (R) | X |  |
|  | [Clozapine versus other atypical antipsychotics for schizophrenia](http://onlinelibrary.wiley.com/doi/10.1002/14651858.CD006633.pub2/abstract) (R) | X |  |
|  | [Antidepressants for people with both schizophrenia and depression](http://onlinelibrary.wiley.com/doi/10.1002/14651858.CD002305/abstract) (R) | X |  |
|  | [Early intervention for psychosis](http://onlinelibrary.wiley.com/doi/10.1002/14651858.CD004718.pub3/abstract) (R) | X |  |
|  | [Antidepressants for smoking cessation](http://onlinelibrary.wiley.com/doi/10.1002/14651858.CD000031.pub4/abstract) (R) | X |  |
|  | [Hyperbaric oxygen therapy for the adjunctive treatment of traumatic brain injury](http://onlinelibrary.wiley.com/doi/10.1002/14651858.CD004609.pub3/abstract) (R) | X |  |
|  | [Mirtazapine versus other antidepressive agents for depression](http://onlinelibrary.wiley.com/doi/10.1002/14651858.CD006528.pub2/abstract) (R) | X |  |
|  | [Preventive interventions for postnatal psychosis](http://onlinelibrary.wiley.com/doi/10.1002/14651858.CD009991.pub2/abstract) (R) | X |  |
|  | [Lithium versus antidepressants in the long-term treatment of unipolar affective disorder](http://onlinelibrary.wiley.com/doi/10.1002/14651858.CD003492.pub2/abstract) (R) | X |  |
|  | [Effectiveness of brief alcohol interventions in primary care populations](http://onlinelibrary.wiley.com/doi/10.1002/14651858.CD004148.pub3/abstract) (R) | X |  |
|  | [Social norms information for alcohol misuse in university and college students](http://onlinelibrary.wiley.com/doi/10.1002/14651858.CD006748.pub3/abstract) (R) | X |  |
|  | [Haloperidol alone or in combination for acute mania](http://onlinelibrary.wiley.com/doi/10.1002/14651858.CD004362.pub2/abstract) (R) | X |  |
|  | [Crisis interventions for people with borderline personality disorder](http://onlinelibrary.wiley.com/doi/10.1002/14651858.CD009353.pub2/abstract) (R) | X |  |
|  | [Cognitive behavioural therapy versus other psychosocial treatments for schizophrenia](http://onlinelibrary.wiley.com/doi/10.1002/14651858.CD008712.pub2/abstract) (R) | X |  |
|  | [Pharmacological treatment for depression during opioid agonist treatment for opioid dependence](http://onlinelibrary.wiley.com/doi/10.1002/14651858.CD008373.pub2/abstract) (R) | X |  |
|  | [Olanzapine IM or velotab for acutely disturbed/agitated people with suspected serious mental illnesses](http://onlinelibrary.wiley.com/doi/10.1002/14651858.CD003729.pub2/abstract) (R) | X |  |
|  | [Amphetamines for schizophrenia](http://onlinelibrary.wiley.com/doi/10.1002/14651858.CD004964/abstract) (R) | X |  |
|  | [Psychological and educational interventions for preventing depression in children and adolescents](http://onlinelibrary.wiley.com/doi/10.1002/14651858.CD003380.pub3/abstract) (R) | X |  |
|  | [Interferon alpha for chronic hepatitis D](http://onlinelibrary.wiley.com/doi/10.1002/14651858.CD006002.pub2/abstract) (R) | X |  |
|  | [Tricyclic drugs for depression in children and adolescents](http://onlinelibrary.wiley.com/doi/10.1002/14651858.CD002317.pub2/abstract) (R) | X |  |
|  | [Family intervention for schizophrenia](http://onlinelibrary.wiley.com/doi/10.1002/14651858.CD000088.pub3/abstract) (R) | X |  |
|  | [Antiviral treatment for chronic hepatitis C in patients with human immunodeficiency virus](http://onlinelibrary.wiley.com/doi/10.1002/14651858.CD004888.pub2/abstract) (R) | X |  |
|  | [Combined pharmacotherapy and psychological therapies for post traumatic stress disorder (PTSD)](http://onlinelibrary.wiley.com/doi/10.1002/14651858.CD007316.pub2/abstract) (R) | X |  |
|  | [Individual psychodynamic psychotherapy and psychoanalysis for schizophrenia and severe mental illness](http://onlinelibrary.wiley.com/doi/10.1002/14651858.CD001360/abstract) (R) | X |  |
|  | [Collaborative care approaches for people with severe mental illness](http://onlinelibrary.wiley.com/doi/10.1002/14651858.CD009531.pub2/abstract) (R) | X |  |
|  | [Hypnosis during pregnancy, childbirth, and the postnatal period for preventing postnatal depression](http://onlinelibrary.wiley.com/doi/10.1002/14651858.CD009062.pub2/abstract) (R) | X |  |
|  | [Interventions for preventing relapse and recurrence of a depressive disorder in children and adolescents](http://onlinelibrary.wiley.com/doi/10.1002/14651858.CD007504.pub2/abstract) (R) | X |  |
| Interpersonal Violence | Hydroxyethyl starch (HES) versus other fluid therapies: effects on kidney function (R) | X |  |
|  | Interventions for protecting renal function in the perioperative period (R) | X |  |
|  | [Pool fencing for preventing **drowning** of children](http://onlinelibrary.wiley.com/doi/10.1002/14651858.CD001047/abstract) (R) | X |  |
|  | [Home safety education and provision of safety equipment for injury prevention](http://onlinelibrary.wiley.com/doi/10.1002/14651858.CD005014.pub3/abstract) (R) | X |  |
|  | [Aerosolized prostacyclin for acute lung injury (ALI) and acute respiratory distress syndrome (ARDS)](http://onlinelibrary.wiley.com/doi/10.1002/14651858.CD007733.pub2/abstract) (R) | X |  |
|  | [Social norms information for alcohol misuse in university and college students](http://onlinelibrary.wiley.com/doi/10.1002/14651858.CD006748.pub3/abstract) (R) | X |  |
|  | [Antenatal psychosocial assessment for reducing perinatal mental health morbidity](http://onlinelibrary.wiley.com/doi/10.1002/14651858.CD005124.pub2/abstract) (R) | X |  |
| Exposure to Forces of Nature | [Antifibrinolytics for heavy menstrual bleeding](http://onlinelibrary.wiley.com/doi/10.1002/14651858.CD000249/abstract) (R) | X |  |
|  | [Mosquito larval source management for controlling malaria](http://onlinelibrary.wiley.com/doi/10.1002/14651858.CD008923.pub2/abstract) (R) | X |  |
|  | [Physician anaesthetists versus non-physician providers of anaesthesia for surgical patients](http://onlinelibrary.wiley.com/doi/10.1002/14651858.CD010357.pub2/abstract) (R) | X |  |
|  | [Antibiotic prophylaxis for leptospirosis](http://onlinelibrary.wiley.com/doi/10.1002/14651858.CD007342.pub2/abstract) (R) | X |  |
|  | [Amniotomy for shortening spontaneous labour](http://onlinelibrary.wiley.com/doi/10.1002/14651858.CD006167.pub4/abstract) (R) | X |  |
| Collective Violence and Legal Intervention | Interventions for promoting reintegration and reducing harmful behaviour and lifestyles in street-connected children and young people | X |  |
|  | Vitamin C for preventing and treating pneumonia (R) | X |  |
|  | Prophylactic antibiotics for penetrating abdominal trauma (R) | X |  |
